# In-depth analysis of FeNi-based nanoparticles for the oxygen evolution reaction

**DOI:** 10.1038/s41598-025-92720-3

**Published:** 2025-03-11

**Authors:** Heydar Habibimarkani, Sarah-Luise Abram, Ana Guilherme Buzanich, Carsten Prinz, Mario Sahre, Vasile-Dan Hodoroaba, Jörg Radnik

**Affiliations:** Federal Institute for Materials Research and Testing (BAM), Unter den Eichen 87, 12205 Berlin, Germany

**Keywords:** Electrochemistry, Surface chemistry, Nanoscale materials

## Abstract

This study investigates the effect of varying iron-to-nickel ratios on the catalytic performance of Fe-Ni oxide nanoparticles (NPs) for the oxygen evolution reaction (OER). Addressing the issue of high energy wastage due to large overpotentials in OER, we synthesized and characterized different NP catalysts with different Fe: Ni oxide ratios. Transmission Electron Microscopy (TEM), Energy Dispersive X-ray Spectroscopy (EDS), and X-ray Diffraction (XRD) were employed to determine the morphology, elemental and phase composition of the NPs. Furthermore, in-depth profiling with X-ray Photoelectron Spectroscopy (XPS) and Hard X-ray Photoelectron Spectroscopy (HAXPES) revealed that iron predominantly exists as oxide, while nickel exhibits both metallic and oxidic forms depending on the Fe content. XPS indicated an enrichment of iron at the NP surface, whereas HAXPES and EDS data agreed on the bulk stoichiometry. The assessment of the catalytic activity via cyclic voltammetry (CV) showed that the Fe: Ni ratio of 2:3 exhibited superior performance, characterized by lower overpotential and a smaller Tafel slope.

**Supplementary Information:**

The online version contains supplementary material available at 10.1038/s41598-025-92720-3.

## Introduction

The rising energy demand and the depletion of natural resources have initiated a revolutionary era centered on finding earth-abundant energy alternatives and creating efficient energy storage devices. The recent surge in climate change underscores the need for extensive research into carbon-neutral and efficient energy solutions^1–4^. Hydrogen is recognized as a clean, renewable fuel free of carbon, which can be produced through electrochemical water splitting that can solve this problem^5^. Water splitting comprises two half-reactions: hydrogen evolution reaction (HER) at the cathode, oxygen evolution reaction (OER) at the anode. However, the overall reaction efficiency is limited. A major challenge is the slow kinetics of the four-electron OER, which demands a significantly higher applied potential than the thermodynamic standard potential^6,7^.

Efficient electrocatalysts are needed to improve the slow kinetics of the OER for practical applications. Currently, noble metal-based electrocatalysts like iridium dioxide and ruthenium dioxide are the most effective catalysts used in water electrocatalysis. Nevertheless, these catalysts are plagued by scarcity and high costs, making them unsuitable for use in water splitting industries to produce economical hydrogen energy resources. As a result, significant efforts are being made to develop effective non-noble metal catalysts for OER. Earth-abundant transition metal compounds, such as Ni, Fe, Co, etc., are considered promising candidates for enhancing OER in water electrolysis^8–10^.

FeNi-based catalysts are particularly notable for their high activity at low overpotentials in alkaline conditions, performing at levels comparable to the most efficient noble metal-based catalysts^11,12^. Fe–Ni nanoparticles (NPs) have been synthesized through several physical and chemical routes, including hydrogen plasma reaction^13^, hydrogen reduction of Fe and Ni inorganic salts^14,15^, reverse micelle method^16,17^, hydrothermal reduction^18–21^, sonochemical decomposition^22–24^ and electrodeposition in a flow cell^25,26^.

In 1987, Corrigan was the first to demonstrate the effects of Fe on OER by examining Ni oxide mixed with different ratios of Fe, revealing that the synergistic interaction between Fe and Ni significantly enhanced OER activity. By precipitating Fe (10% at. to 50% at.) in a composite iron/nickel hydrous oxide, the Tafel slope was greatly lowered (by ~ 65%), indicating faster reaction kinetics for OER^27^. Louie et al. found that electrodeposited Fe-Ni films with 40% Fe exhibit significantly higher OER activity than pure Ni or Fe films. The optimal activity was linked to changes in stability and redox properties of Ni in the films^28^. Suryanto et al. synthesized Ni–Fe NPs using various oleate complexes and examined the electrochemical activities of the overall water-splitting processes. The study presents a Janus NP catalyst of nickel and iron oxide, excelling in both HER and OER. Its superior performance is due to the strong electronic coupling at the nickel–iron oxide interface, offering a cost-effective alternative to platinum-based catalysts^29^. Among transition metal-based materials, spinel nanostructure oxides like NiFe_2_O_4_ exhibit excellent electrocatalytic properties, including high electrical conductivity, reactivity, and superior durability for OER applications^30^. For instance, Liu et al. reported using mesoporous 1D NiFe_2_O_4_ as an electrode material for OER studies and found that NiFe_2_O_4_ nanorods with a surface area of 165.9 m^2^/g exhibited an overpotential of 342 mV at 10 mA/cm^2^ with a Tafel slope of 44 mV/dec^31^. In another work, nanoscale spinel-type Ni–Fe-based oxides were synthesized through a solvothermal method, revealing that the Ni_x_Fe_3−x_O_4_/Ni nanocomposite with an x value around 0.36 showed the highest OER activity. This nanocomposite achieved an electrocatalytic current density of 10 mA/cm^2^ at an overpotential of 225 mV, with a Tafel slope of 44 mV/dec in an alkaline electrolyte^32^.

A recent study on a Ni modified Fe_3_O_4_ single crystal surface has highlighted the benefits of Fe-Ni mixed oxides for oxygen evolution and has pinpointed an optimal composition range for (Fe, Ni)Ox, identifying the Fe: Ni ratio of 2:3 as having the best OER performance^33^. this has confirmed the results described for powders^34^. However, they lack detailed chemical characterization of the catalyst surface and bulk, which could help understand the mechanisms driving the improved activity. Resolving these questions could pave the way for the creation of new materials with superior oxygen evolution activity.

To address these questions, we synthesized various Fe-Ni oxide NPs with different relative compositions, ranging from pure iron containing to pure nickel containing NPs. These NPs were characterized using advanced characterization methods, including X-ray Photoelectron Spectroscopy (XPS), Hard X-ray Photoelectron Spectroscopy (HAXPES), Energy Dispersive X-Ray Spectroscopy (EDS), and Transmission Electron Microscopy (TEM), which allowed us to gain detailed insights into the morphology and the surface and bulk chemistry of the NPs.

## Methods

### Synthesis of pure and mixed metal oxide NPs

**Fig. 1:**
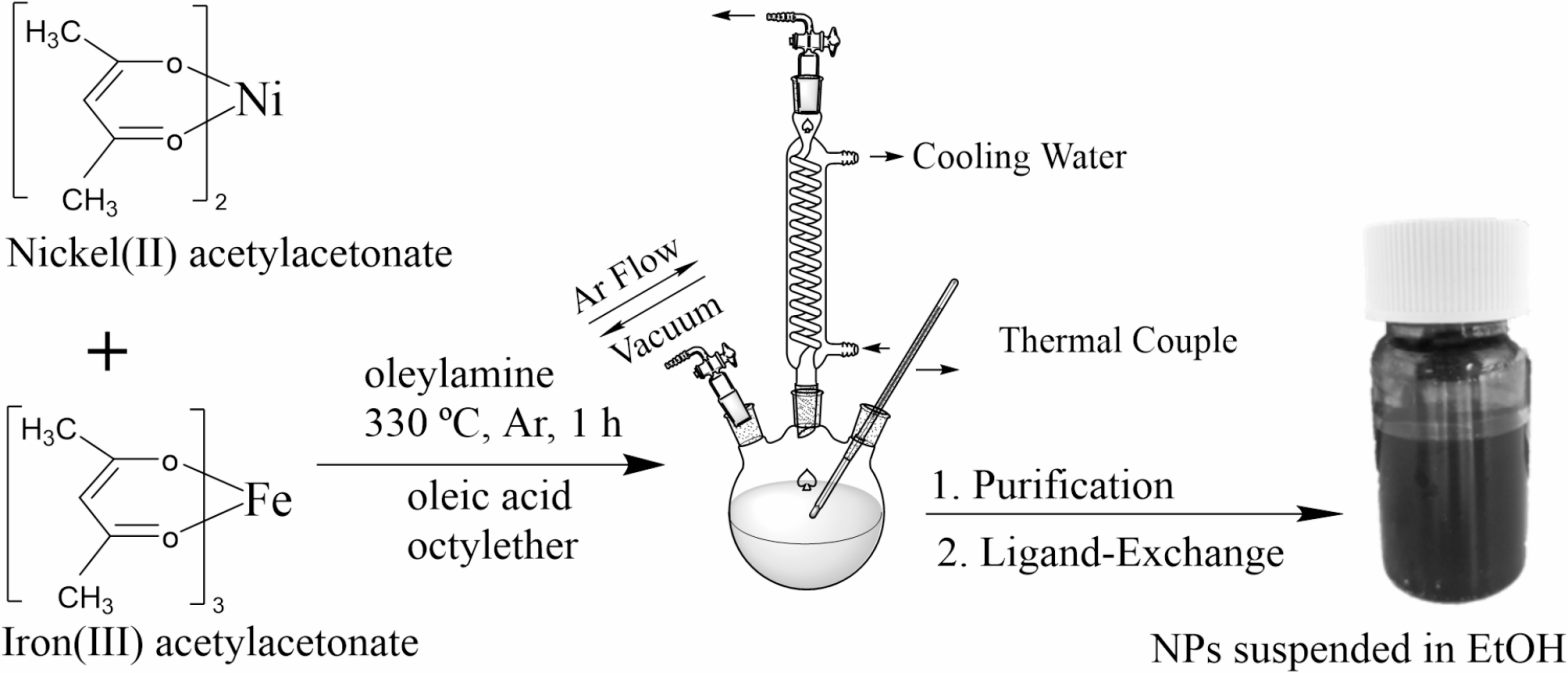
Synthesis Procedure of Fe-Ni-O NPs with different ratios Fe: Ni.

The synthesis procedure for pure iron oxide and nickel oxide nanoparticles (NPs), as well as for those with varied Fe: Ni atom-% ratios (3:1, 2:1, 3:2, 1:1, 2:3) are illustrated in Fig. 1. Nickel (II) acetylacetonate (3:1; 0.412 mmol, 2:1; 0.55 mmol, 3:2; 0.65 mmol, 1:1; 0.825 mmol, 2:3; 1 mmol) and Iron (III) acetylacetonate (3:1; 1.237 mmol, 2:1; 1.1 mmol, 3:2; 1 mmol, 1:1; 0.825 mmol, 2:3; 0.65 mmol) and for pure iron oxide NPs, Iron (III) acetylacetonate (1.65 mmol), for pure nickel oxide NPs, Nickel (II) acetylacetonate (1.65 mmol), along with oleic acid (6.31 mmol), oleylamine (12.16 mmol), and octylether (6.86 mmol) were combined in a 50 mL three-necked round-bottom flask. The mixture was gradually heated to 330 °C at a rate of 6 °C/min under magnetic stirring (310 rpm) and an argon flow of 0.1 L/min. After stirring the mixture for 1 h, it was allowed to cool to room temperature. The NPs were precipitated from the reaction mixture by the addition of ethanol under ambient conditions, purified by three washing steps in toluene/ethanol mixtures, and finally redispersed in toluene^35^.

### Surface ligand exchange

The NPs, already dispersed in toluene, were purified by adding ethanol, which caused the NPs to precipitate. The precipitated NPs were then redispersed in hexane. A 10 mL hexane dispersion containing about 10 mg/mL of NPs was combined with a 10 mL solution of NOBF_4_ (nitrosonium tetrafluoroborate) in dichloromethane, at a concentration of 0.01 M at room temperature. The mixture was gently shaken until the NPs precipitated, (ca. 5 min). After centrifugation, the precipitated NPs were redispersed in N, N-dimethylformamide (DMF) and purified by precipitation with a 1:1 mixture of toluene and hexane and subsequent centrifugation. The precipitate was redispersed in DMF to create a colloidally stable dispersion.

Poly(vinyl pyrollidone) (PVP, molecular weight 40,000) was used to further stabilize the NPs. 200 mg of PVP were added to 20 mL of the NPs dispersion in DMF (ca. 10 mg/mL), and vigorously stirred for 40 min. After precipitation of the NPs with acetone, they were redispersed in ethanol, forming a stable dispersion^36^.

### Catalyst ink Preparation

The catalyst films were formed by applying a dispersion of the catalyst (2.2 mg) onto a glassy carbon (GC) electrode with a 6 mm diameter. This dispersion was made by mixing the catalyst with 49 µL of the NPs dispersed in ethanol, 49 µL of Milli Q water, and 2 µL of Nafion. The resulting ink was ultrasonicated for 30 min at 25 °C to ensure homogeneity. Then, two aliquots of 10 µL each were placed onto the electrode surface and left to dry at room temperature for 15 min. The electrodes were then transferred to a desiccator under vacuum conditions, covering a geometric area of 0.28 cm^2^ (calculated using a diameter of 6 mm for GC)^37^.

## Cyclic voltammetry (CV)

CV experiments were conducted at room temperature in a three-electrode system set within a glass cell (Metrohm, Herisau, Switzerland). The setup included a glassy carbon counter electrode and an Ag/AgCl electrode immersed in 3 M KCl as the reference electrode. The working electrode was also made of glassy carbon, coated with the catalyst, and immersed in an electrolyte of 1 M KOH with a pH of 13.6. Electrochemical measurements were performed using a Metrohm Autolab PGSTAT302N potentiostat controlled by Nova Software, while data analysis was facilitated by Origin software. Electrode potentials were measured against the Ag/AgCl reference electrode and standardized to the reversible hydrogen electrode (RHE) scale using the Nernst equation as follows:

1 $$\:{E}_{RHE}\:=\:{E}_{Ag/AgCl}\:+\:0.197\:V\:+\:0.059\:V\:\times\:\:{pH}_{electrolyte}$$

where E_RHE_ represents the converted potential vs. RHE, E_Ag/AgCl_ is the obtained potential vs. Ag/AgCl, and 0.197 V is the electrode potential of Ag/AgCl at room temperature. Tafel slopes were derived from the relationship between the overpotential (η) and the logarithm of the current density (log j), following the equation:

2 $$\:\eta\:\:=\:\text{b}\:\text{l}\text{o}\text{g}\:j\:+\:\text{a}$$

where b represents the Tafel slope (mV/dec), η the overpotential calculated as η = E_RHE_ − 1.23 V, and a is the intercept of Tafel plot. These values were obtained from the kinetically controlled region of the CV curve to assess the electrocatalytic performance at a scan rate of 10 mV/s.

### X-ray diffraction (XRD)

A 5 µL drop of the nanoparticle suspension in ethanol was drop-cast onto a cleaned silicon wafer and allowed to dry at room temperature. To ensure the formation of a uniform and continuous layer of NPs, this process was repeated ten times on the same area of the wafer. The prepared samples were then analyzed using XRD and XPS to assess the structural and chemical characteristics of the deposited NPs.

XRD measurements were carried out using a Seifert XRD 3000 TT diffractometer (Ahrensburg, Germany) equipped with a Cu Kα tube. The device operated at an electron acceleration voltage of 40 kV and a current of 40 mA. The measurements were conducted with angle steps of 0.05° and a measurement time of 4 s per step. The experiments were performed in a grazing incidence configuration (GIXRD) with an incident angle Ω = 2°, using an X-ray beam collimated by a Göbel mirror.

### Transmission electron microscopy (TEM) and energy-dispersive X-ray spectroscopy (EDS)

NPs suspended in ethanol were diluted at a ratio 1:10 (1 part NPs suspension to 10 parts ethanol) and vigorously shaken before being drop-cast (10 µL) onto clean gold grids, which were positioned on filter paper. The ethanol was allowed to evaporate at ambient temperature, facilitating a uniform distribution of NPs on the grid. After evaporation, the grids were stored in a contamination-free environment for at least one day prior to the measurements^38^.

TEM images were obtained using a Talos F200S transmission electron microscope (Thermo Fisher Scientific) operated at 200 kV. A Ceta 16 M camera (TEM mode) and HAADF (High-Angle Annular Dark-Field) and BF (Bright Field) detectors (STEM mode; Scanning TEM) were used to capture the images. A Super-X G2 X-ray detection system equipped with two silicon drift detectors was used in conjunction with STEM for EDS analysis to determine the elemental composition of the samples, with an acquisition time of 60 s. Velox software Version 3.3 was used for imaging and evaluation.

### X-ray photoelectron spectroscopy (XPS) and hard X-ray photoelectron spectroscopy (HAXPES)

XPS and HAXPES analyses were conducted using the ULVAC-PHI “Quantes” spectrometer (Chanhassen, USA). This instrument features two X-ray sources: a monochromatic Al Kα source emitting at 1486.6 eV for XPS and a monochromatic Cr Kα source emitting at 5414.8 eV for HAXPES. This setup allowed for XPS and HAXPES measurements to be taken precisely at the same point on the sample. The X-ray beam spot size was adjusted to 100 μm for these experiments. Photoelectrons were gathered at a 45° emission angle for both techniques. The Al Kα source was positioned with its X-ray beam perpendicular to the sample surface, while the incident angle of the X-ray beam emitted by the Cr Kα source was set at 22°. The vacuum within the sample chamber was maintained below 10^− 6^ Pa throughout the experiments. Analyses were conducted on three distinct areas of each sample, utilizing low energy electrons and Ar^+^ ions for charge neutralization^39^. For the quantitative analysis, the survey spectra were used, which were measured with a pass energy of 280 eV and a step size of 1 eV. The percentage composition (in at-%) was determined from the peak areas after subtracting Shirley backgrounds and using relative sensitivity factors provided by the manufacturer with MultiPaK. PHI MultiPak Software Version 9.9.2 was used for the quantification of the atomic concentration using the automatic peak indexing routine. Peaks that were not correctly found or were mislabelled were added or deleted manually. The relative empirical sensitivity factors (RSF) were provided by the manufacturer PHI. The binding energy (BE) scale was calibrated according to a PHI procedure that uses binding energy data from ISO 15,472^40^. The intensity was calibrated with the PHI MultiPaK software based on an idea of Seah^41^. A Shirley background was used for the quantification. For the peak fitting with the Unifit software, the PHI datasets had to be converted from SPE to NPL format. For the peak fitting, Unifit 2024 (Unifit-Software, Leipzig, Germany) was employed using a sum of Gaussian-Lorentzian curves. All the measurements were performed with a 100 μm spot at three different positions.

## Results and discussions

### Electrochemical measurements

**Fig. 2:**
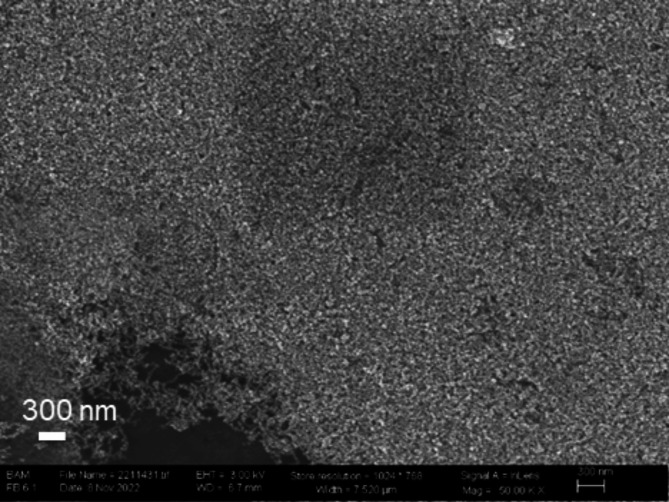
SEM image of the NPs Fe: Ni = 2:3 on glassy carbon after the drop casting.

CV measurements were performed on NPs with varying nominal Fe: Ni at%-ratios between 3:1 and 2:3 as well as pure Ni and Fe containing NPs. To verify the success of the catalyst ink preparation and the coverage of the glassy carbon electrode, SEM images were taken. Figure 2 presents an SEM image depicting NPs with a Fe: Ni = 2:3 spread over a glassy carbon substrate. The image reveals a compact and thick coverage of the electrode surface. The Nafion ionomer acts as both a stabilizer and a binder within the catalyst-solvent inks, resulting in a surface free of NP agglomerates and marked by uniformity of the deposited ink as a critical attribute for accurate cyclic voltammetry measurements. The beneficial effects of Nafion on catalyst performance and surface area uniformity have been documented in the literature^42,43^.

**Fig. 3:**
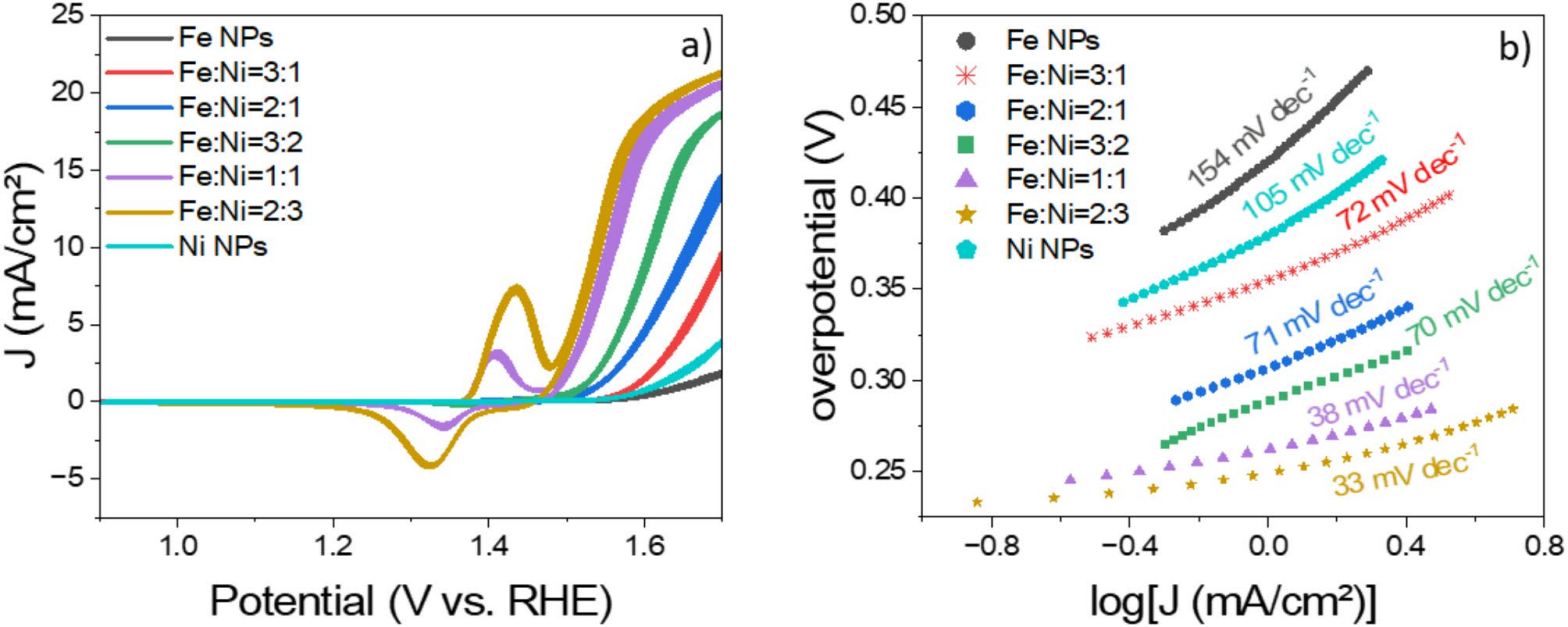
(**a**) Cyclic voltammograms of the Fe-Ni materials with different atomic ratios, in 1 M KOH, Ar atmosphere, and at a scan rate 10 mV/s, (**b**) Corresponding Tafel slopes.

Figure 3; Table 1 present the electrochemical performance of pure iron oxide (Fe NPs), nickel oxide (Ni NPs), and various nominal Fe: Ni ratios (3:1, 2:1, 3:2, 1:1, 2:3) using cyclic voltammetry (CV) and Tafel slope analysis.

Figure 3a shows the cyclic voltammograms acquired in 1 M KOH under an Ar atmosphere with a scan speed of 10 mV/s. The overpotential needed to achieve a specific current density is an important catalytic parameter for assessing the energy efficiency of integrated (photo-)electrochemical water-splitting systems and comparing the performance of catalyst materials. Pure Fe and Ni NPs exhibit higher overpotentials compared to the various Fe: Ni mixed samples. The Fe: Ni ratio 3:1 (shown in red) requires an overpotential of 430 mV at a current density of 5 mA/cm^2^. As the Ni content increases, the activity toward the OER improves. The OER overpotentials for Fe: Ni ratios 2:1, 3:2, and 1:1 are 380, 340, and 300 mV at 5 mA/cm^2^, and 430, 370, and 320 mV at 10 mA/cm^2^, respectively. The Fe: Ni ratio 2:3 exhibits the lowest overpotential of 310 mV to achieve a current density of 10 mA/cm^2^ and reaches 280 mV at 5 mA/cm^2^, representing a reduction of ~ 150 mV compared to the Fe: Ni ratio 3:1. Upon magnification of the plots for redox peaks across all samples, it was observed that peaks around 1.4 V are present in all Ni-containing samples, indicating that these peaks correspond to the oxidation of Ni(II) to Ni(III). In contrast, this redox feature is absent in the pure Fe oxide sample, confirming that the peak is characteristic for nickel oxidation and not associated with iron^44^.

Figure 3b presents Tafel slopes extracted from the CV data plotted in Fig. 3a. The pure Fe and Ni NPs display Tafel slopes of 154 mV/dec and 105 mV/dec, respectively, whereas the various-mixed Fe: Ni samples show values ranging from 33 to 72 mV/dec. The Fe: Ni = 2:3 ratio exhibits a Tafel slope of 33 mV/dec, suggesting favorable OER kinetics and efficient electron transfer, and the best electrochemical performance out of all catalyst materials tested in this study. Other Fe: Ni ratios and pure-metal oxides show higher overpotentials and Tafel slopes, indicating less efficient catalytic processes.

Table 1 provides a summary of the overpotentials required to reach current densities of 5 and 10 mA/cm^2^, along with the corresponding Tafel slopes for each Fe: Ni ratio. The Fe: Ni mixed samples demonstrate improved OER performance with increasing Ni content, with the Fe: Ni = 2:3 ratio showing the best catalytic efficiency. In contrast, the Fe-rich nanoparticles with a Fe: Ni = 3:1 ratio shows the worst efficiency among the mixed samples.

**Fig. 4:**
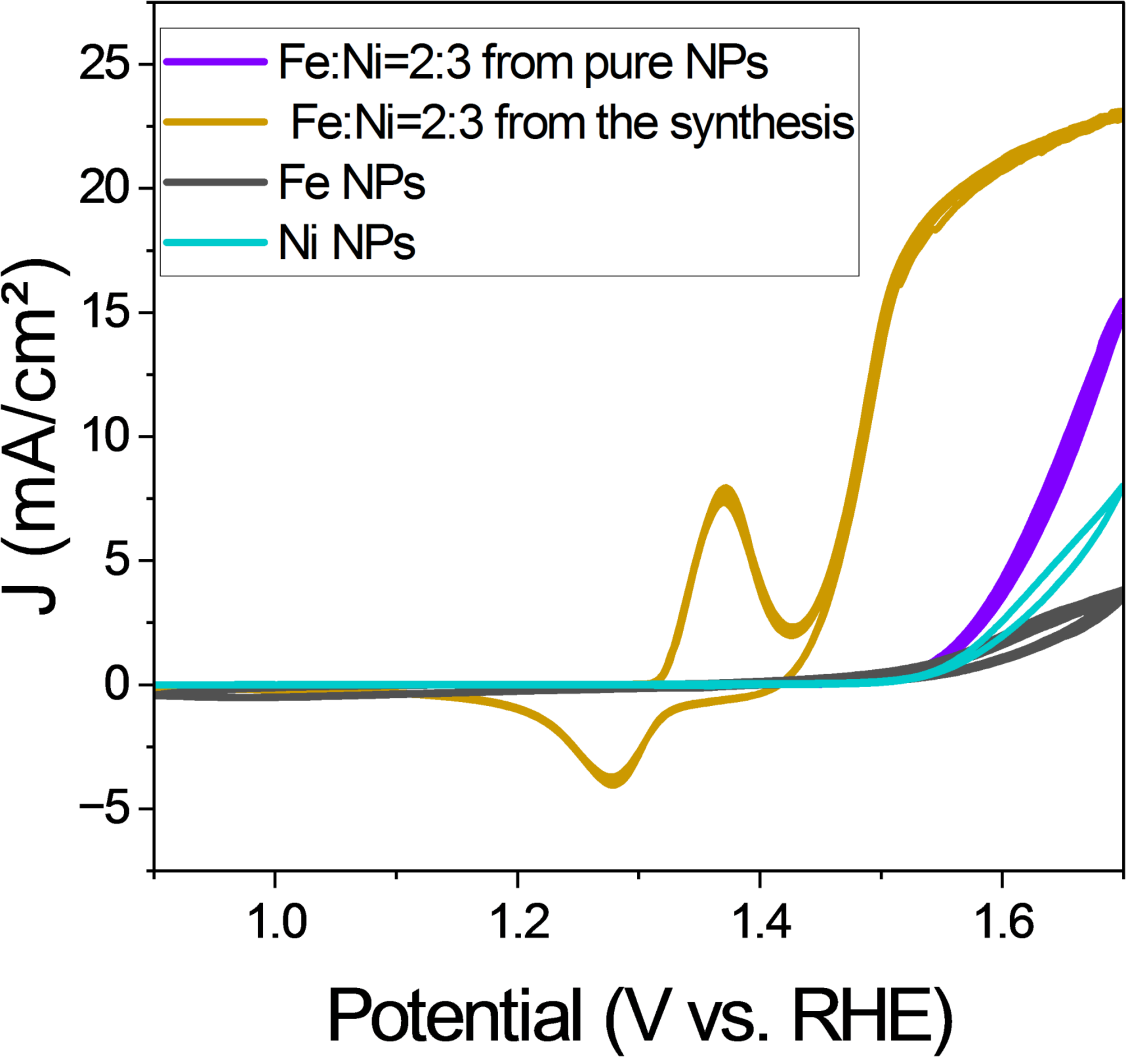
Cyclic voltammograms of NPs with a nominal Fe: Ni ratio of 2:3 obtained from the pure NPs and prepared in the synthesis. The CVs of the pure NPs are shown for comparison.

In all, the different Fe: Ni oxide NP catalysts were more efficient than the pure Fe or Ni NPs. In a further experiment we compared the performance of the NPs with the best catalyst efficiency with a mixture of pure Fe and Ni containing NPs with the same Fe: Ni ratio of 2:3 (Fig. 4). This mixture of the pure Fe and Ni NPs showed a slightly lower overpotential than the pure NP with addition of the other component, but a significantly worse efficiency than the best one with the same Fe: Ni ratio.

To better understand the catalytic performance of the synthesized NPs, we have investigated the crystallographic phases of the NPs with the different nominal compositions. For a detailed analysis four materials were selected: pure iron oxide, pure nickel oxide, the most effective catalyst Fe: Ni = 2:3, and the least effective catalyst Fe: Ni = 3:1. The morphology and size of these selected NPs, were analyzed as well as surface and bulk chemical composition by TEM, EDS, XPS, and HAXPES.

**Table 1:** OER performance of synthesized NPs with different nomimal Fe: Ni ratios and the corresponding pure NPs.

Sample	Overpotential vs. RHE (mV) at j = 5 mA/cm^2^	Overpotential vs. RHE (mV) at j = 10 mA/cm^2^	Tafel slope (mV/dec)
Fe	> 430	> 430	154 ± 9
Fe: Ni = 3:1	430 ± 4	> 430	72 ± 2
Fe: Ni = 2:1	380 ± 4	430 ± 7	71 ± 2
Fe: Ni = 3:2	340 ± 3	370 ± 8	70 ± 2
Fe: Ni = 1:1	300 ± 4	320 ± 7	38 ± 1
Fe: Ni = 2:3	280 ± 4	310 ± 5	33 ± 1
Ni	> 430	> 430	105 ± 4

### XRD analysis

**Fig. 5:**
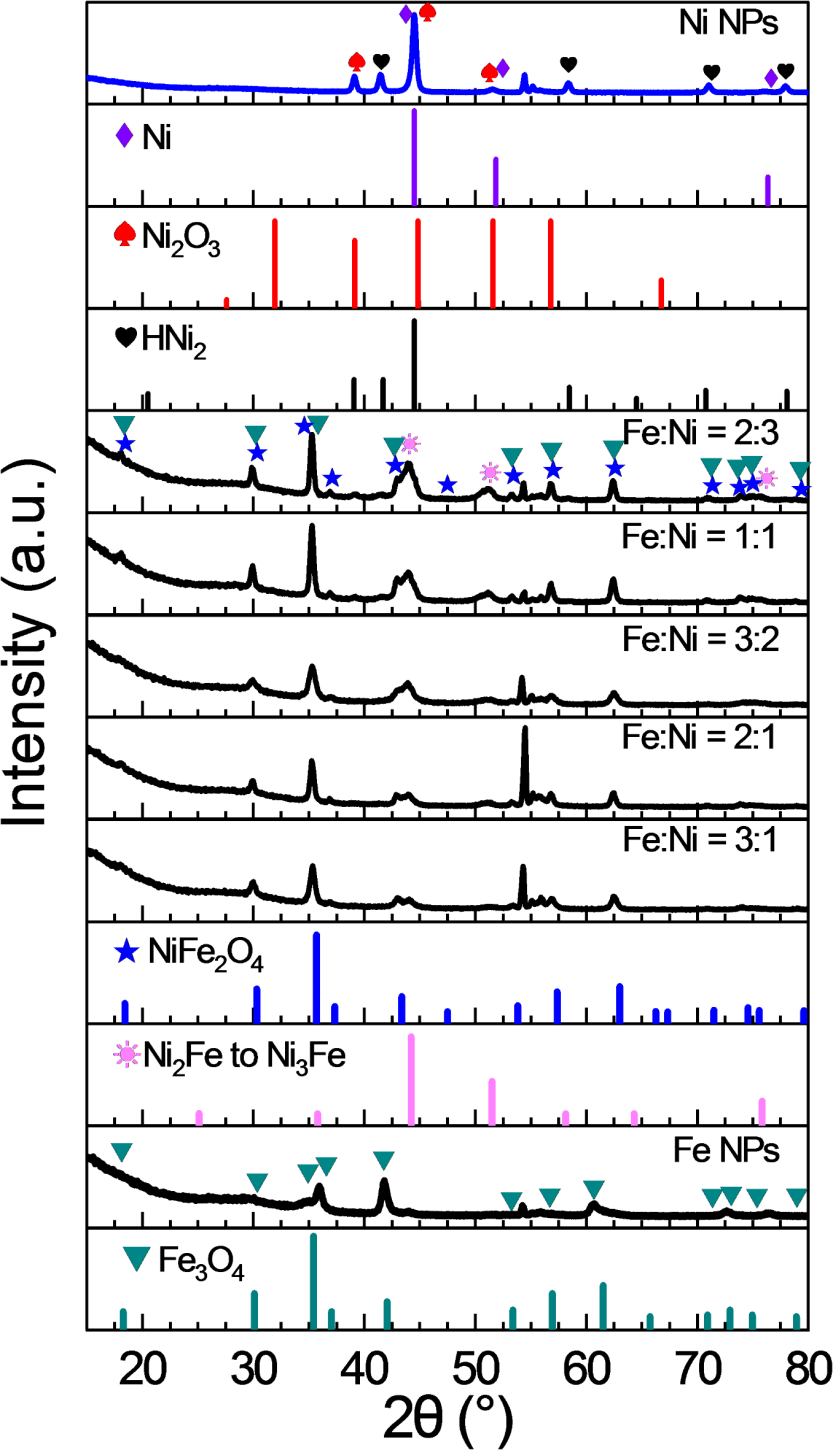
XRD patterns of NPs showing peaks for the iron oxides magnetite or maghematite (green triangles ), Trevorite (NiFe_2_O_4_) (blue asterisks ), Awaruite (Ni_2_Fe to Ni_3_Fe) (pink sun symbols ), nickel hydride (black hearts ), nickel oxide Ni_2_O_3_ (red spade ), Nickel (purple diamond ).

The X-ray diffraction (XRD) analysis, as shown in Fig. 5, reveals insights into the crystallographic phase composition of the synthesized NPs with varying nominal Fe: Ni ratios. For the pure iron oxide NPs only a single phase corresponding to pure iron oxide was identified. It is difficult to clearly distinguished between magnetite (Fe_3_O_4_) and maghemite (γ-Fe_2_O_3_) as both have nearly similar cubic crystal systems^45^.

For the pure nickel containing sample, the XRD pattern suggests the presence of both metallic nickel and nickel oxide phases in agreement with the ICDD data of (ICDD no PDF 4–850) and (ICDD no PDF 14–481), respectively^46^. Further, a hydride phase of nickel (ICDD no PDF 33–606) is also indicated. Based on our synthesis route, the formation of nickel hydride seems unlikely. More likely is the possibility of a slight change in the crystal lattice of the Ni nanoparticles compared to bulk Ni.

The NPs with varied Fe: Ni ratios, namely 3:1, 2:1, 3:2, 1:1, and 2:3, exhibit a trevorite (NiFe_2_O_4_) phase (), which is structurally similar to magnetite. According to the Fe-Ni-O phase diagram^47,48^, spinel phases are stable across a wide range of Fe/Ni ratios and at elevated temperatures. Therefore, the XRD results are consistent with the phase diagram, which predicts spinel formation in Fe-Ni-O systems. This phase was also observed for the mixed particles. A slight shift to lower 2Ɵ values was observed for an increased Ni amount, which can be explained with the increasing incorporation of Ni into the cubic lattice. The trevorite structure is also cubic and belongs to the same space group Fm3̅m, with a lattice constant slightly larger, i.e. 8.48 Å, which aligns with the substitution of Ni in the lattice^49^.

Moreover, marked peaks () denote the presence of Awaruite (ranging from Ni_2_Fe to Ni_3_Fe) as a Ni-rich Ni-Fe alloy according to the JCPDS# 88-1715^50^. It is noteworthy to mention that the XRD peaks at 2θ ~ 54° present in all samples are attributed to the to the (311) peak of the Si, related to the Si wafer substrate^51^.

The XRD results provide a clear confirmation of the formation of magnetite in the case of pure iron oxide, a mixture of metallic and oxide phases for pure nickel NPs, and a mixture of oxide and Ni rich alloys for the Fe-Ni oxide NPs.

### TEM analysis

**Fig. 6:**
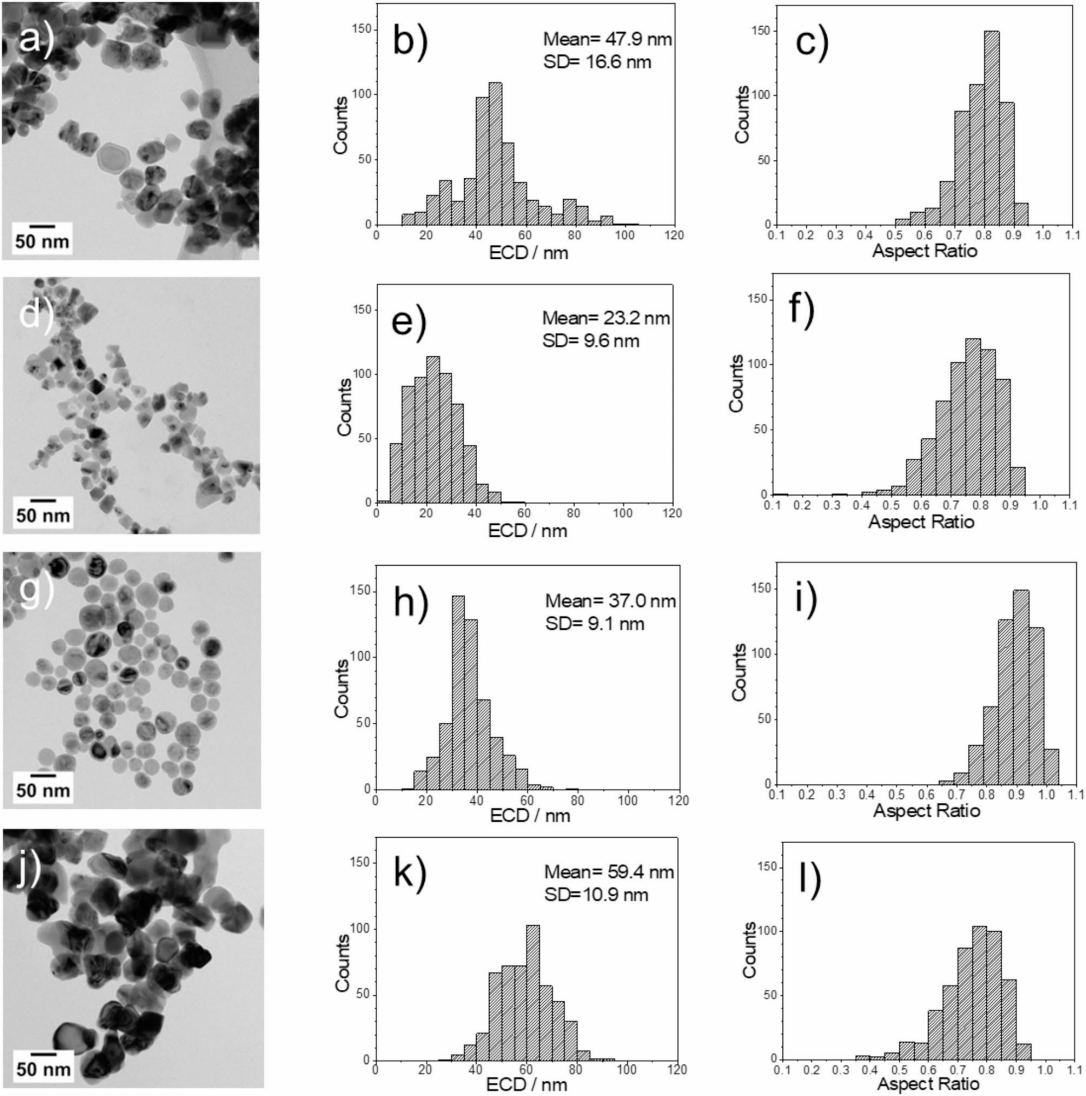
TEM analysis of the NP size and shape. (**a**–**c**) Fe: Ni = 2:3 (TEM image, ECD, aspect ratio), (**d**–**f**) Fe: Ni = 3:1 (TEM image, ECD, aspect ratio), (**g**–**i**) Iron NPs (TEM image, ECD, aspect ratio), (**j**–**l**) Nickel NPs (TEM image, ECD, aspect ratio).

Figure 6 presents a quantitative analysis of the four selected NP materials: the two different iron-nickel oxide NPs with varying stoichiometries (Fe: Ni = 2:3 and Fe: Ni = 3:1) with the best and worse catalytic performance, and the two sets of the pure metal oxide NPs (iron and nickel). The area Equivalent Circular Diameter (ECD) and aspect ratio of approximately 500 particles of each type were calculated using TEM images and ImageJ software^52^ to assess their size distribution, shape, and morphological characteristics as routinely applied by us for oxide NPs^53^.

The ECD provides a measure of the effective diameter of a particle as if it was a circle, offering insights into the size of the NPs. The aspect ratio, calculated as the ratio of the Min Feret to the Max Feret diameter, indicates the particle shape, with values closer to 1 indicating more spherical shapes.

The Fe: Ni = 2:3 NPs exhibited a mean ECD of 47.9 nm with a standard deviation (SD) of 16.6 nm, as shown in Fig. 5b. The distribution of the aspect ratios (Fig. 6c), which is close to a value of one, suggests that most of the NPs are rather spherical in shape rather than elongated. The Fe: Ni = 3:1 NPs presented a smaller mean ECD of 23.2 nm and an SD of 9.6 nm, as shown in Fig. 6e, with a broad aspect ratio distribution (Fig. 6f) suggesting a diverse range of shapes.

The pure iron oxide NPs (Fig. 6h) have a mean ECD of 37.0 nm and an SD of 9.1 nm, and their aspect ratio distribution pointed towards a spherical tendency, see Fig. 6i. The pure nickel oxide NPs presented a higher mean ECD of 59.4 nm and an SD of 10.9 nm (Fig. 6k). Their aspect ratio histogram was more evenly spread (Fig. 6l), indicating a diversity in particle shape which is similar to that of Fe: Ni = 3:1 (Fig. 6f).

The particle size distribution of the various iron-rich NPs appears to closely resemble that of the pure iron NPs (Fig. 6e and h). Similarly, despite different mean values, the distribution pattern for the various nickel-rich NPs aligns with that of the pure nickel NPs (Fig. 6b and k). Observations from the particle morphology and aspect ratios indicate that all particles generally exhibit a circular shape with aspect ratios slightly lower than 1. Whereas for all Ni containing NPs the maximum of the aspect ratio was between 0.7 and 0.8, the FeO_x_ NPs with a maximum at 0.9 showed the most pronounced spherical morphology (Fig. 6i).

**Fig. 7:**
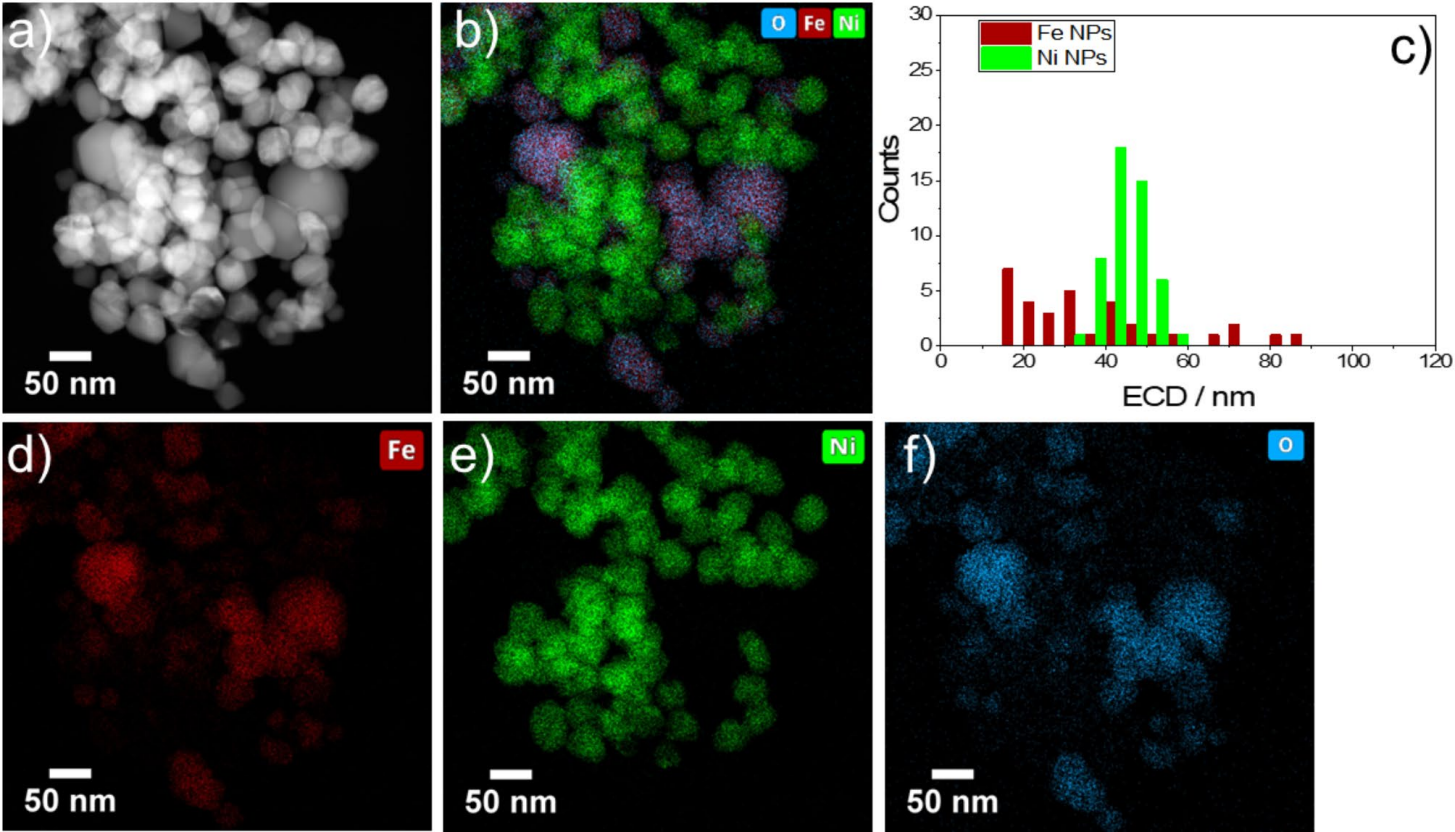
Fe: Ni = 2:3 NPs: (**a**) HAADF-STEM image, (**b**) EDS elemental mapping of O K, Fe K and Ni K X-ray lines, (**c**) Histogram of the size distribution of Fe and Ni NPs as detected by EDS in (**b**), separate EDS elemental mappings of Fe (**d**), Ni (**e**), and O (**f**).

**Fig. 8:**
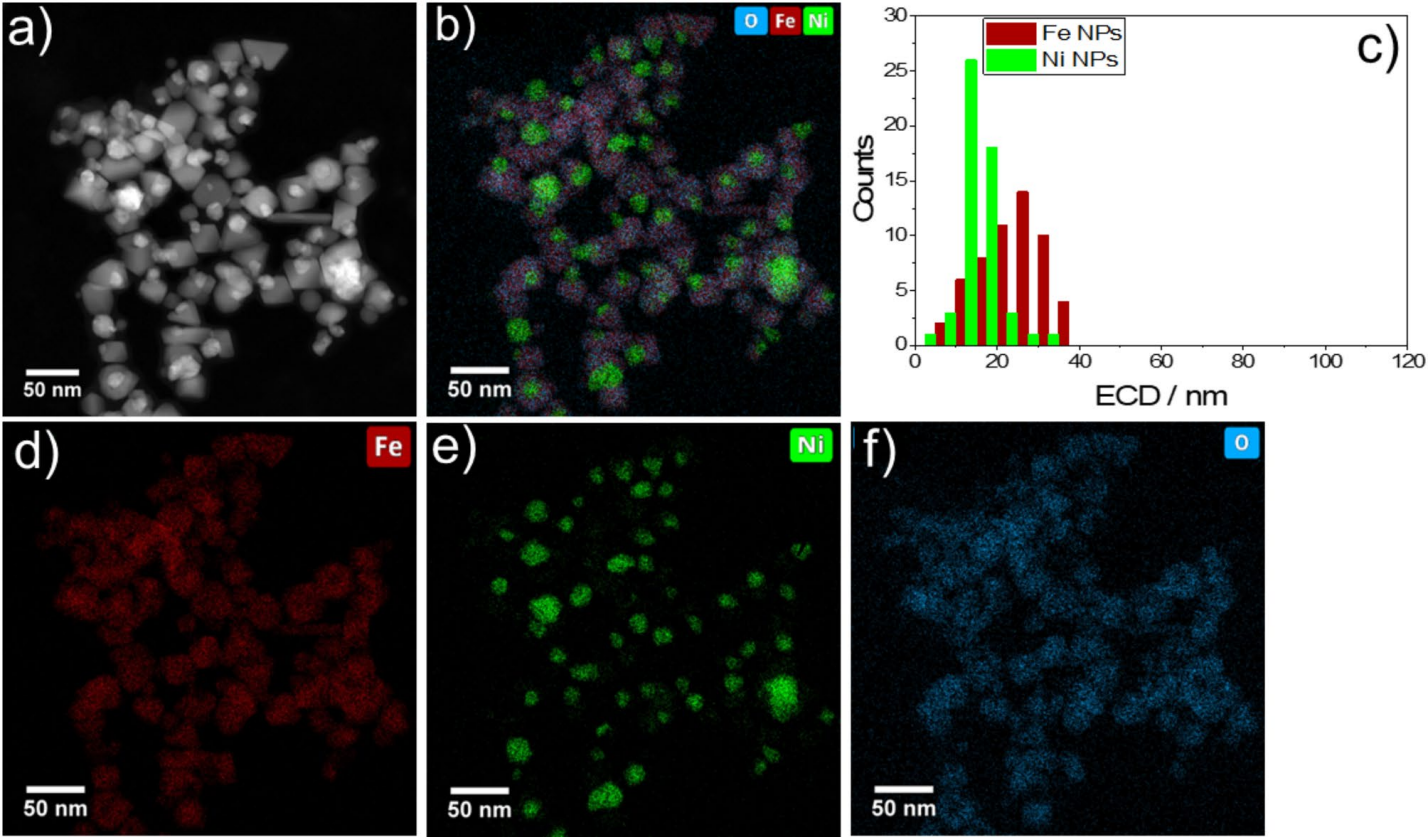
Fe: Ni = 3:1 NPs: (**a**) HAADF-STEM image, (**b**) EDS elemental mapping of O K, Fe K and Ni K X-ray lines, (**c**) Histogram of the size distribution of Fe and Ni NPs as detected by EDS in (**b**), (**d**) separate EDS elemental mappings of Fe (**d**), Ni (**e**), and O (**f**).

Figures 7 and 8 provide a comprehensive analysis through HAADF-STEM and EDS elemental mapping of the two selected Fe: Ni NP samples with nominal Fe: Ni ratios of 2:3 and 3:1, respectively. The HAADF images (Figs. 7a and 8a) reveal good contrast, which enables to clearly see the differences in composite structures and the spatial arrangement of the elements. This helps to distinguish between the compositional structures of the two samples. The corresponding EDS maps (Figs. 7b and 8b) were used to extract the size distribution of Fe, (in red), and Ni (green), providing a qualitative visualization of the elemental distribution within each NP sample. The particle size histograms elucidate a consistent size range within each iron-rich (Fe: Ni = 3:1) and nickel-rich (Fe: Ni = 2:3) materials. The Fe: Ni material with a ratio of 3:1 (Fig. 8c) exhibits smaller particle sizes as well as a narrower size distribution for Fe NPs, suggesting a higher uniformity in the Fe NP size - as opposed to the slightly broader size distribution observed for the Fe: Ni = 2:3 material (Fig. 7c). Additionally, the pure Ni particles within the Fe: Ni = 3:1 sample appear to be dispersed as small(er) particles throughout the sample.

A closer examination of the Fe EDS maps between the two samples reveals a significant difference: for the nominal Fe: Ni ratio of 3:1, Fe is distributed more evenly across the sample, whereas, for the nominal Fe: Ni ratio of 2:3, Fe is concentrated in specific regions, indicating a degree of segregation or clustering within the sample matrix.

Although the Fe: Ni = 3:1 NPs have a smaller size, the Fe: Ni = 2:3 NPs with higher nickel content demonstrate superior performance. This can be attributed to the optimal balance of nickel and iron in the 2:3 sample, which enhances electronic conductivity and provides more active sites for the catalytic reaction. The increased nickel content also improves the material’s intrinsic activity, effectively compensating for the larger particle size. This favorable composition and distribution lead to more efficient catalyst utilization and better overall performance^33^. This indicates that particle size as in our study does not affect the catalytic performance.

In both Fig. 7 (d, e,f) and Fig. 8 (d, e,f), the elemental mapping separately highlights the spatial distribution of iron (Fe), nickel (Ni), and oxygen (O). It is evident that all three elements are distributed throughout each sample, with certain areas enriched with Fe or Ni. It must be noted that the enrichment of O correlated with Fe, indicating that the vast majority of Fe was oxidic. In contrast, a significant portion of Ni appeared to be metallic. All in all, this high-resolution mapping emphasizes the varying stoichiometric ratios of Fe and Ni within the NPs. To estimate the integral Fe: Ni ratio three different regions of each sample with agglomerates ranging from a few hundred nanometers to a few micrometers were measured with EDS as shown in Figures S1, S2, and Tables S1, S2 in the Supplementary Information. These measurements confirm that it was possible to synthesize the samples with nominal Fe: Ni ratios of 3:1 and 2:3.

### XPS/HAXPES

**Fig. 9:**
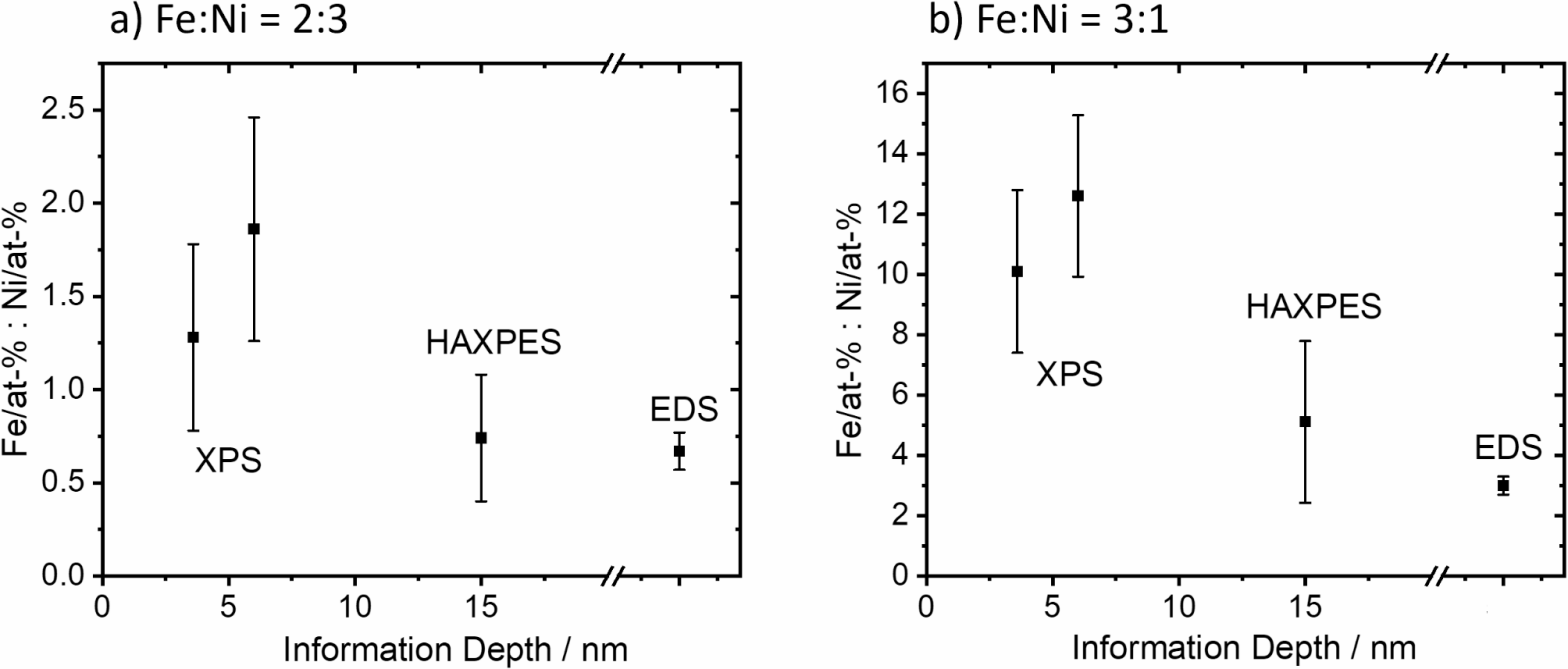
Comparative analysis of Fe: Ni atom-% ratios measured using XPS, HAXPES, and EDS for nanoparticulate materials with nominal Fe: Ni ratios of (**a**) 3:1 and (**b**) 2:3. The information depth corresponds to three times the inelastic mean free path, which was calculated using the JTP formula^55^.

As mentioned above, EDS confirmed the nominal Fe: Ni ratios of 3:1 and 2:1 for both samples. Additionally, XPS and HAXPES measurements were carried out in order to determine the Fe: Ni atom-% ratios. These methods probe varying depths of the material, i.e. XPS ~ 10 nm, HAXPES ~ 30 nm, STEM/EDS in the range of about 100 nm, offering insight into the elemental distribution both at the surface and in the bulk.The Fe: Ni atom-% ratios determined through different analytical methods (XPS, HAXPES, and STEM/EDS) are represented in Fig. 9 for two different compositions, Fe: Ni = 3:1 and Fe: Ni = 2:3.

The *Ni LMM* Auger peak overlaps with the Fe *2p*_*3/2*_ peak in the XPS region. For both XPS and HAXPES, the Fe *2p*_*1/2*_ and *Ni 2p*_*1/2*_ peaks were used to avoid any influence of the Fe *2s* on the Ni *2p*_*3/2*_ quantification. Furthermore, the Fe *3p* and Ni *3p* peaks were used for the determination of the Fe: Ni ratio with XPS with a higher information depth than the *2p* peaks obtained with the same X-ray energy. XPS, the most surface-sensitive method of these three, shows a higher Fe: Ni ratio for both samples compared to HAXPES and EDS. The ratios obtained with the *2p* peaks are slightly lower than for the *3p* peaks. This indicates a higher Ni content in the region in the outermost layers than in the deeper layers. All in all, in the near-surface region an enrichment of Fe was observed for both samples, while HAXPES and EDS data agree on their values. The agreement of the EDS and HAXPES results can be expected for the sizes of the NPs with a mean diameter below 50 nm for the mixed particles. This confirms through both EDS and HAXPES that the nominal composition corresponds to the real composition. The main sources of uncertainty are sample preparation, the relative sensitivity factors used, and the determination of peak areas, especially for small peaks. It should be noted, that information depth can be determined with a relative uncertainty between 15% and 20%^54^.

**Fig. 10:**
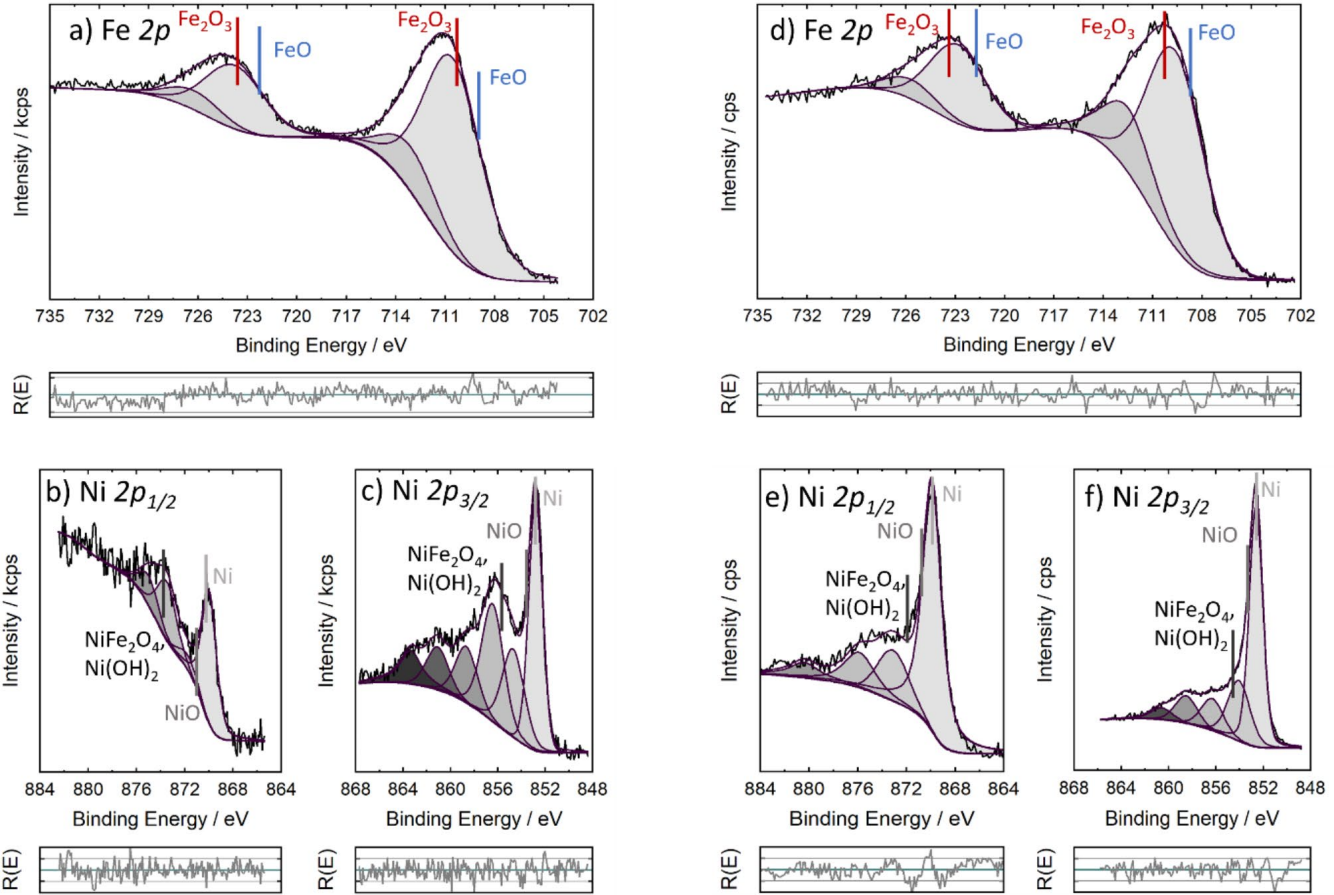
XPS (**a**–**c**) and HAXPES (**d**–**f**) spectra of Fe: Ni = 2:3 NPs (**a**) XPS: Fe *2p* region. (**b**, **c**) XPS: Ni *2p*_1/2_ and Ni *2p*_3/2_ region, respectively. (**d**) HAXPES: Fe *2p* region. (**e**, **f**) HAXPES: Ni *2p*_1/2_ and Ni *2p*_3/2_ region, respectively. The reference binding energies of the Fe *2p* are from^60^, and those for the Ni *2p* peaks are from^61^ .For Fe3O4 and Fe_2_O_3_ the same binding energies were observed.

**Fig. 11:**
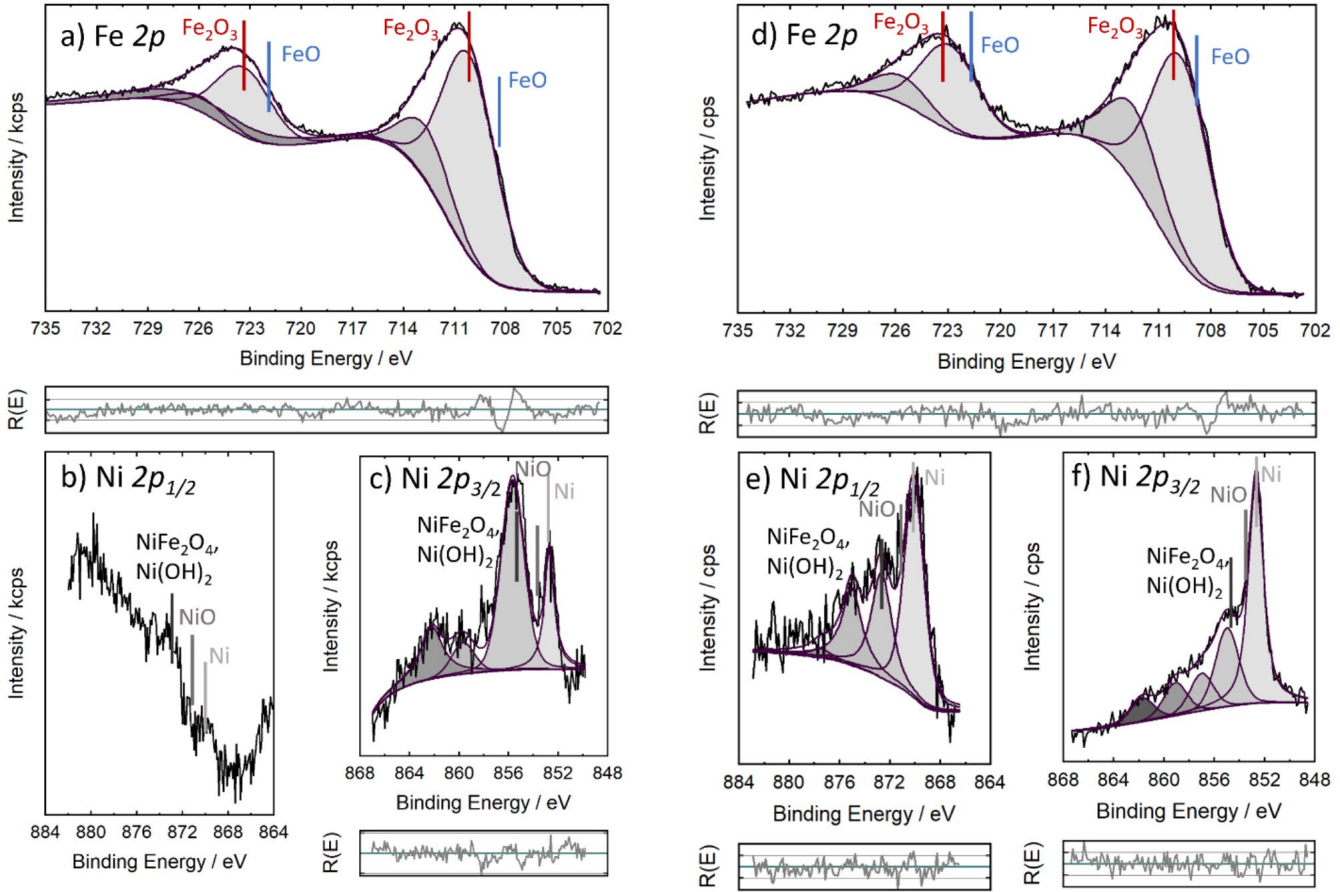
XPS (**a**–**c**) and HAXPES (**d**–**f**) spectra of Fe: Ni = 3:1 NPs (**a**) XPS: Fe *2p* region. (**b**, **c**) XPS: Ni *2p*_1/2_ and Ni *2p*_3/2_ region respectively. (**d**) HAXPES: Fe *2p* region. (**e**, **f**) HAXPES: Ni *2p*_1/2_ and Ni *2p*_3/2_ region respectively. Same references like for Fig. 11 were used^60.61^.

High-resolution XPS and HAXPES exhibit some differences in the valence states of Fe and Ni (Figs. 10 and 11). For iron, only oxidic components were found for both XPS and HAXPES, with a binding energy of 710.6 eV for the Fe *2p*_*3/2*_ photoelectrons and 723.8 eV for the Fe *2p*_*1/2*_ photoelectrons in XPS. These binding energies hint to Fe (3+) as major Fe component^56^. With HAXPES, slightly lower binding energy was found which could indicate to bivalent Fe as minor component. In contrast, Ni shows both metallic Ni with a Ni *2p*_*3/2*_ binding energy of 852.8 eV and oxidic Ni with a Ni *2p*_*3/2*_ binding energy of 855.7 eV, which can be correlated to bivalent Ni in form of Ni(OH)_2_ or trivalent Ni in NiFe_2_O_4_. The satellite peaks typical for bivalent Ni with *2p*_*3/2*_ binding energies between 860 eV and 865 eV were measured for all spectra. On the other hand, mixed NiFe_2_O_4_ was detected with XPS. The satellite peaks typical for bivalent Ni with *2p*_*3/2*_ binding energies between 860 eV and 865 eV were measured for all spectra. For the Fe-rich NPs, the Ni *2p*_*1/2*_ binding energies cannot be determined due to the influence of the Fe LM_23_M_23_ peak around 890 eV. For the Ni-rich sample and the HAXPES spectra it was possible to detect the corresponding Ni *2p*_*1/2*_ peaks^57^. For comparison, the corresponding spectra of the pure Fe and Ni NPs are shown in the Supporting Information.

**Fig. 12:**
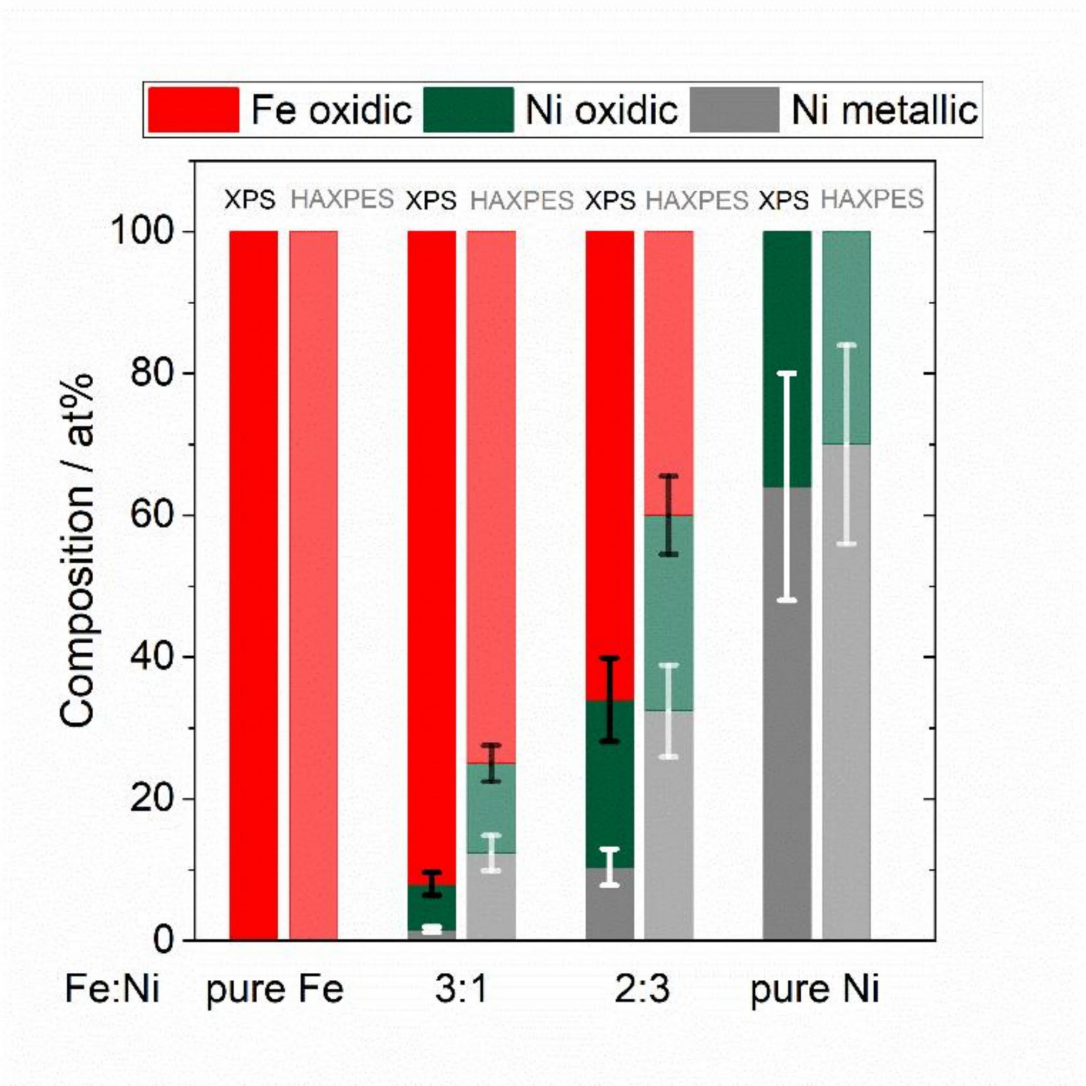
Comparison of oxidic Fe, oxidic Ni and metallic Ni compositions with XPS and HAXPES. For the determination of oxidic and metal Ni the Ni *2p*_*3/*2_ peaks were considered.

HAXPES showed a higher amount of metallic Ni than XPS, indicating that metallic Ni is preferentially present in the bulk, see Fig. 12. Furthermore, FeO_x_ which is preferentially located in the near-surface region seems to stabilize the oxidic Ni. For the Fe-rich NPs only a negligible amount of metallic Ni was found with XPS. These results indicate that predominantly oxides of iron and nickel are present in the near-surface region, probably as mixed oxides. In contrast, the best performing catalyst with a Fe: Ni ratio of 2:3 shows a significant percentage of metallic Ni in the near surface region with XPS. It is still unclear whether this higher proportion of metallic Ni or the higher amount of oxidic Ni is responsible for the good performance of this nanocatalyst. The XPS/HAXPES results agree with the XRD findings showing Fe-rich oxide phase and Ni-rich metallic phases. In addition, the results are consistent with the EDS mapping results, which showed a correlation between O and Fe.

There are three possible mechanisms for the OER process: the Adsorbate Evolution Mechanism (AEM), Lattice Oxygen Mechanism (LOM), and Oxide Path Mechanism (OPM)^58^. In our system, we suggest that the LOM is the predominant mechanism for all four samples tested, i.e. Fe/Ni ratio of 3:1, Fe/Ni ratio of 2:3, pure iron, and pure nickel. Notably, the overpotential values calculated in our system are close to those observed in the referenced articles, supporting our conclusion. The enhanced performance of the Fe = 2:3 sample is attributed to a favorable balance between Ni and Fe, which promotes lattice oxygen activation and results in improved catalytic efficiency^59^.

## Conclusions

To examine the impact of varying iron to nickel ratios on OER activity and overall catalytic performance, seven different NP catalysts, i.e. five different Fe: Ni oxide ratios, and two pure Fe and Ni oxide materials, were synthesized and analyzed. Combined EDS and HAXPES measurements on two selected NPs showed that the nominal composition was achieved during synthesis. The best performance for the OER characterized by a lower overpotential showed the nanocatalyst with a Fe: Ni ratio of 2:3, which agrees with reported results^33,34^. A mixture with the same Fe: Ni ratio made from the pure Fe and Ni NPs showed a significantly worse performance, which is only slightly better than that of the pure NPs. This observation underlines the importance of intermixed phases for the catalytic performance of the NPs. Although such intermixed Fe-Ni phases were observed with XRD, mapping with EDS showed a distinct regions of Fe- and Ni-rich regions. Surprisingly, this varation was more pronounced for the best mixed catalyst with Fe: Ni of ratio 2:3 than for the worst one. The chemical characterization methods XPS and HAXPES were used to perform an in-depth analysis of the NPs. This revealed that the majority of Fe is trivalent. In contrast, Ni is present in both oxidic and metallic forms. A comparison of the XPS and HAXPES results showed that both iron oxide and Ni oxide are enriched at the surface, probably as mixed oxides. The distribution of oxide Fe-rich and metal Ni-rich regions found with EDS probably reflects the bulk composition. Nevertheless, for the NPs with the Fe: Ni ratio 2:3 and the best performance, a significant amount of metallic Ni was detected in the near-surface region. Whether this proportion of metallic Ni or the higher amount of oxidic Ni is responsible for the good electrocatalytic properties of these NPs is the subject of further investigation. In summary, these studies show the importance of combining surface-sensitive methods such as XPS, methods with a high information depth in the nanoparticle range like HAXPES and spatially resolved methods such as TEM coupled with EDS mapping to gain a better understanding of such nanoparticulate systems.

## Electronic supplementary material

Below is the link to the electronic supplementary material.

Supplementary Material 1

## Data Availability

Data are availabe under 10.5281/zenodo.14975965.

## References

[1] Jiao, S., Fu, X., Wang, S. & Zhao, Y. Perfecting electrocatalysts via imperfections: Towards the large-scale deployment of water electrolysis technology. *Energy Environ. Sci.* **14**, 1722–1770. 10.1039/D0EE03635H (2021).

[2] Kudo, A. & Miseki, Y. Heterogeneous photocatalyst materials for water splitting. *Chem. Soc. Rev.* **38**, 253–278. 10.1039/B800489G (2009). 19088977 10.1039/b800489g

[3] Liang, Y., Li, Y., Wang, H. & Dai, H. Strongly coupled inorganic/nanocarbon hybrid materials for advanced electrocatalysis. *J. Am. Chem. Soc.* **135**, 2013–2036. 10.1021/ja3089923 (2013). 23339685 10.1021/ja3089923

[4] Jacobson, T. A. et al. Direct human health risks of increased atmospheric carbon dioxide. *Nat. Sustain.* **2**, 691–701. 10.1038/s41893-019-0323-1 (2019).

[5] Zhang, L. N. et al. Advanced hydrogen evolution electrocatalysts promising sustainable hydrogen and chlor-alkali co-production. *Energy Environ. Sci.* **14**, 6191–6210. 10.1039/D1EE02798K (2021).

[6] Zeng, K. & Zhang, D. Recent progress in alkaline water electrolysis for hydrogen production and applications. *Prog. Energy Combust. Sci.* **36**, 307–326. 10.1016/j.pecs.2009.11.002 (2010).

[7] Ehsan, M. A. et al. Highly effective electrochemical water oxidation by millerite-phased nickel sulfide nanoflakes fabricated on Ni foam by aerosol-assisted chemical vapor deposition. *Energy Fuels*. **35**, 16054–16064. 10.1039/C8NR10138H (2021).

[8] Han, L., Dong, S., & Wang, E. Transition-metal Co, Ni, and Fe)‐based electrocatalysts for the water oxidation reaction. *Adv. Mater.* **28**, 9266–9291. 10.1002/adma.201602270 (2016). 27569575 10.1002/adma.201602270

[9] Li, Y. et al. Ternary NiCoP nanosheet arrays: an excellent bifunctional catalyst for alkaline overall water splitting. *Nano Res.* **9**, 2251–2259. 10.1007/s12274-016-1112-z (2016).

[10] Zhang, J. Y. et al. Anodic hydrazine oxidation assists energy-efficient hydrogen evolution over a bifunctional Cobalt perselenide nanosheet electrode. *Angew. Chem. Int. Ed.* **57**, 7649–7653. 10.1002/anie.201803543 (2018). 10.1002/anie.201803543 29696766

[11] Gong, M. et al. An advanced Ni–Fe layered double hydroxide electrocatalyst for water oxidation. *J. Am. Chem. Soc.* **135**, 8452–8455. 10.1021/ja4027715 (2013). 23701670 10.1021/ja4027715

[12] Trotochaud, L., Ranney, J. K., Williams, K. N. & Boettcher, S. W. Solution-cast metal oxide thin film electrocatalysts for oxygen evolution. *J. Am. Chem. Soc.* **134**, 17253–17261. 10.1021/ja307507a (2012). 22991896 10.1021/ja307507a

[13] Li, X., Chiba, A. & Takahashi, S. Preparation and magnetic properties of ultrafine particles of Fe Ni alloys. *J. Magn. Magn. Mater.* **170**, 339–345. 10.1016/S0304-8853(97)00039-5 (1997).

[14] Suh, Y. J., Jang, H. D., Chang, H., Kim, W. B. & Kim, H. C. Size-controlled synthesis of Fe–Ni alloy nanoparticles by hydrogen reduction of metal chlorides. *Powder Technol.* **161**, 196–201. 10.1016/j.powtec.2005.11.002 (2006).

[15] Wu, H. Q., Cao, Y. J., Yuan, P. S., Xu, H. Y. & Wei, X. W. Controlled synthesis, structure and magnetic properties of Fe1 – xNix alloy nanoparticles attached on carbon nanotubes. *Chem. Phys. Lett.* **406**, 148–153. 10.1016/j.cplett.2005.02.117 (2005).

[16] Ban, I., Drofenik, M. & Makovec, D. The synthesis of iron–nickel alloy nanoparticles using a reverse micelle technique. *J. Magn. Magn. Mater.* **307**, 250–256. 10.1016/j.jmmm.2006.04.010 (2006).

[17] Cushing, B. L., Golub, V. & O’Connor, C. J. Synthesis and magnetic properties of Au-coated amorphous Fe20Ni80 nanoparticles. *J. Phys. Chem. Solids*. **65**, 825–829. 10.1016/j.jpcs.2003.11.027 (2004).

[18] Liao, Q., Tannenbaum, R. & Wang, Z. L. Synthesis of FeNi3 alloyed nanoparticles by hydrothermal reduction. *J. Phys. Chem. B*. **110**, 14262–14265. 10.1021/jp0625154 (2006). 16854130 10.1021/jp0625154

[19] Chen, H., Xu, C., Zhao, G. & Liu, Y. Template-free formation of urchin-like FeNi3 microstructures by hydrothermal reduction. *Mater. Lett.* **91**, 75–77. 10.1016/j.matlet.2012.09.040 (2013).

[20] Chen, Y. C., Zheng, F. C., Min, Y. L., Wang, T. & Zhao, Y. G. Synthesis and properties of magnetic FeNi3 alloyed microchains obtained by hydrothermal reduction. *Solid State Sci.* **14**, 809–813. 10.1016/j.solidstatesciences.2012.04.006 (2012).

[21] Han, T., Xu, C. & Chen, H. Simple synthesis of novel mushroom-like FeNi3 microstructures by a hydrothermal reduction. *Mater. Res. Innovations*. **23**, 39–42. 10.1080/14328917.2017.1362509 (2019).

[22] Lee, E., Park, A. H., Park, H. U. & Kwon, Y. U. Facile sonochemical synthesis of amorphous NiFe-(oxy) hydroxide nanoparticles as superior electrocatalysts for oxygen evolution reaction. *Ultrason. Sonochem.* **40**, 552–557. 10.1016/j.ultsonch.2017.07.048 (2018). 28946457 10.1016/j.ultsonch.2017.07.048

[23] Shafi, K., Gedanken, A., Goldfarb, R. B. & Felner, I. Sonochemical Preparation of nanosized amorphous Fe-Ni alloys. *J. Appl. Phys.* **81**, 6901–6905. 10.1063/1.365250 (1997).

[24] Shafi, K. V. et al. Sonochemical Preparation of nanosized amorphous NiFe2O4 particles. *J. Phys. Chem. B*. **101**, 6409–6414. 10.1021/jp970893q (1997).

[25] Kim, S. H. et al. Effect of saccharin addition on the microstructure of electrodeposited Fe–36 Wt.% Ni alloy. *Surf. Coat. Technol.* **199**, 43–48. 10.1016/j.surfcoat.2004.11.035 (2005).

[26] Li, H. & Ebrahimi, F. Synthesis and characterization of electrodeposited nanocrystalline nickel–iron alloys. *Mater. Sci. Engineering: A*. **347**, 93–101. 10.1016/S0921-5093(02)00586-5 (2003).

[27] Corrigan, D. A. The catalysis of the oxygen evolution reaction by iron impurities in thin film nickel oxide electrodes. *J. Electrochem. Soc.* **134**, 377. 10.1149/1.2100463 (1987).

[28] Louie, M. W. & Bell, A. T. An investigation of thin-film Ni–Fe oxide catalysts for the electrochemical evolution of oxygen. *J. Am. Chem. Soc.* **135**, 12329–12337. 10.1021/ja405351s (2013). 23859025 10.1021/ja405351s

[29] Suryanto, B. H., Wang, Y., Hocking, R. K., Adamson, W. & Zhao, C. Overall electrochemical splitting of water at the heterogeneous interface of nickel and iron oxide. *Nat. Commun.* **10**, 5599. 10.1038/s41467-019-13415-8 (2019). 31811129 10.1038/s41467-019-13415-8 PMC6898202

[30] Feng, Z. et al. Recent progress on NiFe2O4 spinels as electrocatalysts for the oxygen evolution reaction. *J. Electroanal. Chem.* **946**, 117703. 10.1016/j.jelechem.2023.117703 (2023).

[31] Liu, G., Wang, K., Gao, X., He, D. & Li, J. Fabrication of mesoporous NiFe2O4 nanorods as efficient oxygen evolution catalyst for water splitting. *Electrochim. Acta*. **211**, 871–878. 10.1016/j.electacta.2016.06.113 (2016).

[32] Huang, J. et al. Improving electrocatalysts for oxygen evolution using Ni X Fe3–x O4/Ni hybrid nanostructures formed by solvothermal synthesis. *ACS Energy Lett.* **3**, 1698–1707. 10.1021/acsenergylett.8b00888 (2018).

[33] Mirabella, F. et al. Ni-modified Fe3O4 (001) surface as a simple model system for understanding the oxygen evolution reaction. *Electrochim. Acta*. **389**, 138638. 10.1016/j.electacta.2021.138638 (2021).

[34] Dionigi, F. & Strasser, P. NiFe-based (oxy) hydroxide catalysts for oxygen evolution reaction in non‐acidic electrolytes. *Adv. Energy Mater.* **6**, 1600621. 10.1002/aenm.201600621 (2016).

[35] Noh, S. et al. Nanoscale magnetism control via surface and exchange anisotropy for optimized ferrimagnetic hysteresis. *Nano Lett.* **12**, 3716–3721. 10.1021/nl301499u (2012). 22720795 10.1021/nl301499u

[36] Dong, A. et al. A generalized ligand-exchange strategy enabling sequential surface functionalization of colloidal nanocrystals. *J. Am. Chem. Soc.* **133**, 998–1006. 10.1021/ja108948z (2011). 21175183 10.1021/ja108948z

[37] Broicher, C. et al. Iron and manganese containing Multi-Walled carbon nanotubes as electrocatalysts for the oxygen evolution Reaction‐Unravelling influences on activity and stability. *ChemCatChem* **12**, 5378–5384. 10.1002/cctc.202000944 (2020).

[38] Abram, S. L. et al. Iron oxide nanocubes as a new certified reference material for nanoparticle size measurements. *Anal. Chem.* **95**, 12223–12231. 10.1021/acs.analchem.3c00749 (2023). 37566555 10.1021/acs.analchem.3c00749

[39] Chemello, G. et al. Influence of the morphology on the functionalization of graphene nanoplatelets analyzed by comparative photoelectron spectroscopy with soft and hard X-Rays. *Adv. Mater. Interfaces*. **10**, 2300116. 10.1002/admi.202300116 (2023).

[40] 15472 & : IISO Geneva,. (2010).

[41] Seah, M. A system for the intensity calibration of electron spectrometers. *J. Electron Spectrosc. Relat. Phenom.* **71**, 191–204. 10.1016/0368-2048(94)02275-5 (1995).

[42] Li, G. F., Yang, D. & Abel Chuang, P. Y. Defining Nafion ionomer roles for enhancing alkaline oxygen evolution electrocatalysis. *ACS Catal.* **8**, 11688–11698. 10.1021/acscatal.8b02217 (2018).

[43] Morales, D. M., Villalobos, J., Kazakova, M. A., Xiao, J. & Risch, M. Nafion-Induced reduction of manganese and its impact on the electrocatalytic properties of a highly active MnFeNi oxide for bifunctional oxygen conversion. *ChemElectroChem* **8**, 2979–2983. 10.1002/celc.202100744 (2021). 34595088 10.1002/celc.202100744 PMC8457226

[44] Bao, F. et al. Host, suppressor, and promoter—the roles of Ni and Fe on oxygen evolution reaction activity and stability of NiFe alloy thin films in alkaline media. *ACS Catal.* **11**, 10537–10552. 10.1021/acscatal.1c01190 (2021).

[45] Zachariah, M., Aquino, M., Shull, R. & Steel, E. Formation of superparamagnetic nanocomposites from vapor phase condensation in a flame. *Nanostruct. Mater.* **5**, 383–392. 10.1016/0965-9773(95)00260-L (1995).

[46] Praveen, A. E., Ganguli, S. & Mahalingam, V. Prudent electrochemical pretreatment to promote the OER by catalytically inert Iron incorporated metallic Ni nanowires synthesized via the non-classical growth mechanism. *Nanoscale Adv.* **2**, 1927–1938. 10.1039/D0NA00073F (2020). 36132518 10.1039/d0na00073f PMC9418993

[47] Davies, H. & Smeltzer, W. Oxygen and metal activities of the Iron-Nickel‐Oxygen system at 1000° C. *J. Electrochem. Soc.* **119**, 1362. 10.1149/1.2403998 (1972).

[48] Raghavan, V. Fe-Ni-O (iron-nickel-oxygen). *J. Phase Equilib. Diffus.* **31**, 369–371. 10.1007/s11669-010-9714-8 (2010).

[49] Paswan, S. K. et al. Optimization of structure-property relationships in nickel ferrite nanoparticles annealed at different temperature. *J. Phys. Chem. Solids*. **151**, 109928. 10.1016/j.jpcs.2020.109928 (2021).

[50] Qin, S. et al. Synthesis of Ni 4.5 Fe 4.5 S 8/ni 3 S 2 film on Ni 3 Fe alloy foam as an excellent electrocatalyst for the oxygen evolution reaction. *RSC Adv.* **9**, 10231–10236. 10.1039/C9RA00724E (2019). 35520944 10.1039/c9ra00724e PMC9062387

[51] Vaia, R. A., Weathers, M. S. & Bassett, W. A. Anomalous peaks in grazing incidence thin film X-ray diffraction. *Powder Diffr.* **9**, 44–49. 10.1017/S0885715600019679 (1994).

[52] Rasband, W. S. ImageJ (US National Institutes of Health, Bethesda, Maryland, USA, 2011). http://imagej.nih.gov/ij/

[53] Hodoroaba, V.-D., Rades, S. & Unger, W. E. Inspection of morphology and elemental imaging of single nanoparticles by high-resolution SEM/EDX in transmission mode. *Surf. Interface Anal.* **46**, 945–948. 10.1002/sia.5426 (2014).

[54] Radnik, J. et al. Composition, thickness, and homogeneity of the coating of core–shell nanoparticles—possibilities, limits, and challenges of X-ray photoelectron spectroscopy. *Anal. Bioanal. Chem.* **414**, 4331–4345. 10.1007/s00216-022-04057-9 (2022). 35471249 10.1007/s00216-022-04057-9 PMC9142455

[55] Jablonski, A., Tanuma, S. & Powell, C. J. Calculations of electron inelastic mean free paths (IMFPs). XIV. Calculated IMFPs for LiF and Si3N4 and development of an improved predictive IMFP formula. *Surf. Interface Anal.* **55**, 609–637. 10.1002/sia.7217 (2023).

[56] Lee, G. et al. Controlled electrophoretic deposition strategy of binder-free CoFe2O4 nanoparticles as an enhanced electrocatalyst for the oxygen evolution reaction. *ACS Appl. Mater. Interfaces*. **14**, 48598–48608. 10.1021/acsami.2c11456 (2022). 36256595 10.1021/acsami.2c11456

[57] Kesavan, J. K. et al. Ni supported on YSZ: XAS and XPS characterization and catalytic activity for CO2 methanation. *J. Mater. Sci.* **52**, 10331–10340. 10.1007/s10853-017-1179-2 (2017).

[58] Lin, C. et al. In-situ reconstructed Ru atom array on α-MnO2 with enhanced performance for acidic water oxidation. *Nat. Catal.* **4**, 1012–1023. 10.1038/s41929-021-00703-0 (2021).

[59] Li, Y. F. & Selloni, A. Mechanism and activity of water oxidation on selected surfaces of pure and Fe-doped NiO X. *ACS Catal.* **4**, 1148–1153. 10.1021/cs401245q (2014).

[60] Hughes, A., Easton, C., Gengenbach, T., Biesinger, M. & Laleh, M. Interpretation of complex x-ray photoelectron peak shapes. I. Case study of Fe *2p*_*3/2*_ spectra. *J. Vacuum Sci. Technol. A*. 10.1116/6.0003804 (2024).

[61] Biesinger, M. C. et al. Resolving surface chemical States in XPS analysis of first row transition metals, oxides and hydroxides: Cr, Mn, Fe, Co and Ni. *Appl. Surf. Sci.* **257**, 2717–2730. 10.1016/j.apsusc.2010.10.051 (2011).

