# Supplementary material for: In-depth analysis of FeNi-based nanoparticles for the oxygen evolution reaction
Source: Sci Rep. 2025 Mar 11;15:8339. doi: 10.1038/s41598-025-92720-3 (PMC11893910; doi:10.1038/s41598-025-92720-3)
Supplement: Supplementary file 1 — Supplementary Material 1 [file 41598_2025_92720_MOESM1_ESM.docx]

**Supplementary Information**

**In-depth analysis of FeNi-based nanoparticles for the Oxygen Evolution Reaction**

Heydar Habibimarkani, Sarah-Luise Abram, Ana Guilherme Buzanich, Carsten Prinz, Mario Sahre, Vasile-Dan Hodoroaba* and Jörg Radnik*

[*Dan.Hodoroaba@bam.de](mailto:*Dan.Hodoroaba@bam.de); [*Joerg.Radnik@bam.de](mailto:*Joerg.Radnik@bam.de)

Federal Institute for Materials Research and Testing (BAM), Unter den Eichen 87, 12205 Berlin, Germany


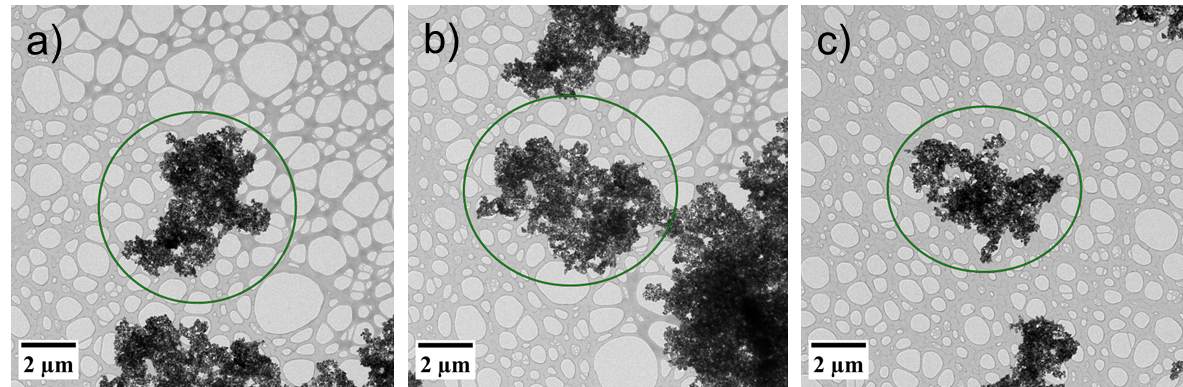


**Figure S1:** TEM images of Fe: Ni= 2:3 sample with EDS analysis regions marked in green circles. (a) Region 1, (b) Region 2, (c) Region 3.

| Sample | EDS | Element | Lines | Atomic Fraction (%) | Atomic Error (%) |
| --- | --- | --- | --- | --- | --- |
| Fe:Ni = 2:3 | Region 1 | Fe | K | 41.3 | 3.9 |
|  |  | Ni | K | 58.7 | 3.9 |
|  | Region 2 | Fe | K | 40.6 | 3.9 |
|  |  | Ni | K | 59.4 | 3.9 |
|  | Region 3 | Fe | K | 42.6 | 3.9 |
|  |  | Ni | K | 57.4 | 3.9 |

**Table S1:** EDS analysis results of Fe: Ni= 2:3 for regions 1, 2, and 3.


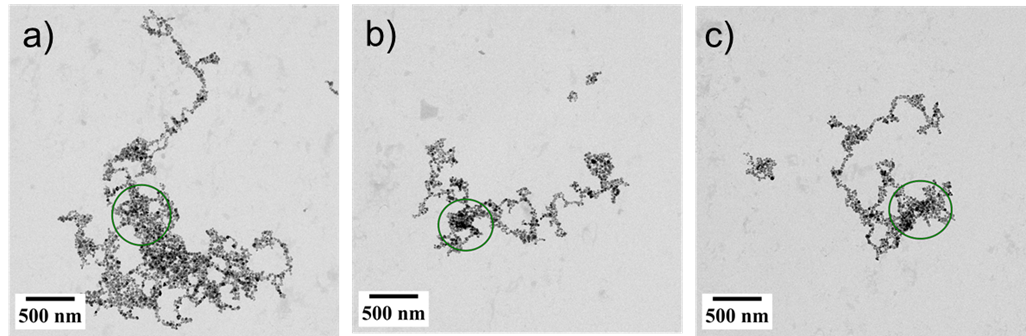


**Figure S2:** TEM images of Fe: Ni= 3:1 sample with EDS analysis regions marked in green circles. (a) Region 1, (b) Region 2, (c) Region 3.

| Sample | EDS | Element | Lines | Atomic Fraction (%) | Error (%) |
| --- | --- | --- | --- | --- | --- |
| Fe:Ni = 3:1 | Region 1 | Fe | K | 72.2 | 4.4 |
|  |  | Ni | K | 27.8 | 4.4 |
|  | Region 2 | Fe | K | 75.3 | 4.0 |
|  |  | Ni | K | 24.7 | 4.0 |
|  | Region 3 | Fe | K | 76.0 | 4.0 |
|  |  | Ni | K | 24.0 | 4.0 |

**Table S2:** EDS analysis results of Fe: Ni= 3:1 for regions 1, 2, and 3.


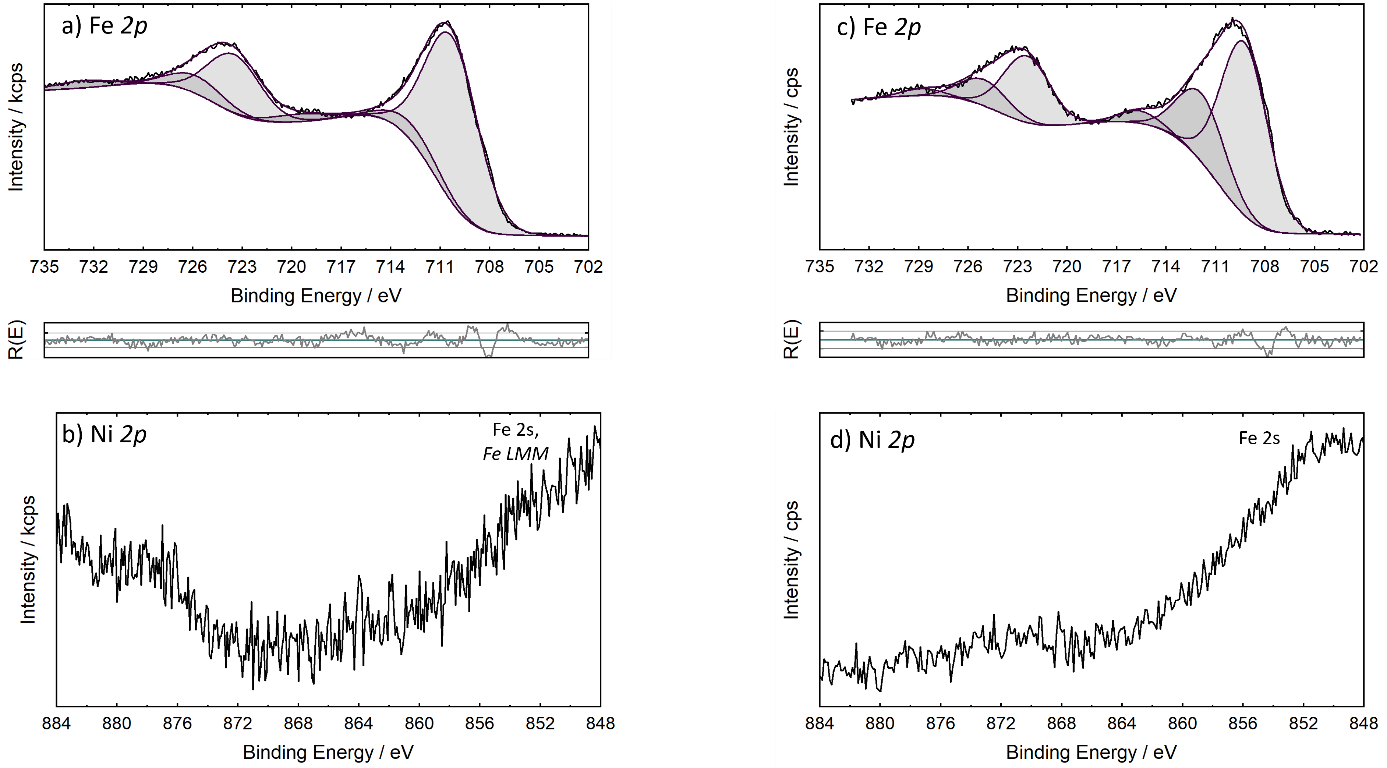


**Figure S3.** XPS (a-b) and HAXPES (c-d) spectra of the pure Fe NPs a) XPS: Fe *2p* region. B) XPS: Ni *2p* region. c) HAXPES: Fe *2p* region. d) HAXPES: Ni *2p* region.


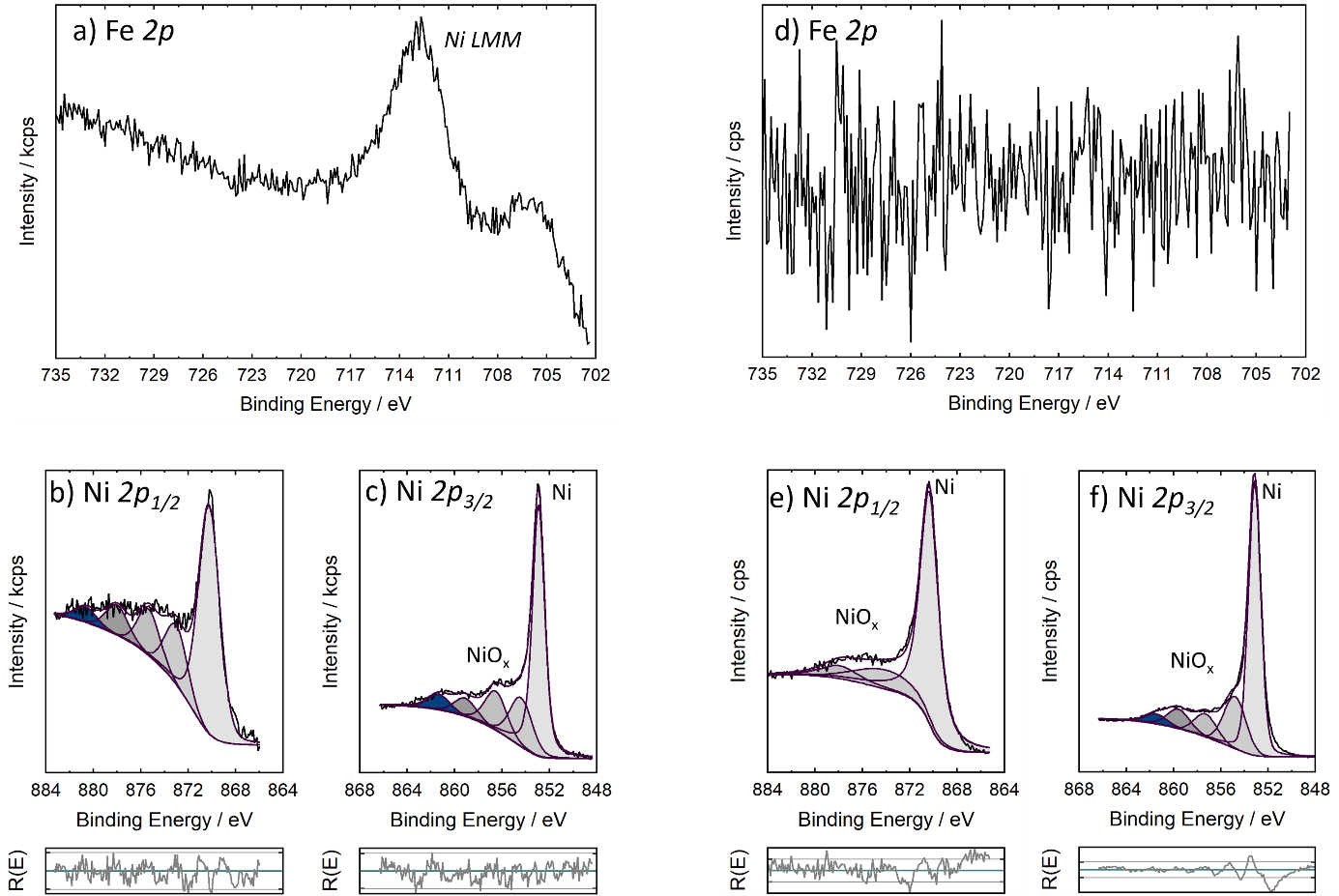


**Figure S4.** XPS (a-c) and HAXPES (d-f) spectra of pure Ni NPs a) XPS: Fe *2p* region. b, c) XPS: Ni *2p*_1/2_ and Ni *2p*_3/2_ region respectively. d) HAXPES: Fe *2p* region. e, f) HAXPES: Ni *2p*_1/2_ and Ni *2p*_3/2_ region respectively.

**Table S3:** XPS/HAXPES fitting parameter of the pure Fe NPs.

XPS:

| Peak | Binding Energy /eV | Height /cps | L-G Mixing | FWHM / eV |
| --- | --- | --- | --- | --- |
| Fe *2p3/2* (1) | 710.3 | 2068 | 0.0 | 3.7 |
| Fe *2p1/2* (1) | 723.5 | 730 | 0.0 | 3.7 |
| Fe *2p3/2* (2) | 710.3 | 209 | 0.0 | 3.9 |
| Fe *2p1/2* (2) | 723.5 | 220 | 0.0 | 3.9 |
| Fe *2p3/2* (3) | 719.5 | 95 | 0.0 | 4.1 |
| Fe *2p1/2* (3) | 732.7 | 89 | 0.0 | 4.1 |

HAXPES:

| Peak | Binding Energy /eV | Height /cps | L-G Mixing | FWHM / eV |
| --- | --- | --- | --- | --- |
| Fe *2p3/2* (1) | 709.3 | 100 | 0.0 | 3.3 |
| Fe *2p1/2* (1) | 722.5 | 39 | 0.0 | 3.3 |
| Fe *2p3/2* (2) | 711.8 | 40 | 0.0 | 3.4 |
| Fe *2p1/2* (2) | 725.0 | 15 | 0.0 | 3.4 |
| Fe *2p3/2* (3) | 715.6 | 7 | 0.0 | 2.9 |
| Fe *2p1/2* (3) | 728.8 | 4 | 0.0 | 2.9 |

**Table S4:** XPS/HAXPES fitting parameter of the Fe:Ni = 3:1 NPs.

XPS:

| Peak | Binding Energy /eV | Height /cps | L-G Mixing | FWHM / eV |
| --- | --- | --- | --- | --- |
| Fe *2p3/2* (1) | 710.1 | 444 | 0.0 | 3.7 |
| Fe *2p1/2* (1) | 723.3 | 124 | 0.0 | 3.4 |
| Fe *2p3/2* (2) | 712.6 | 444 | 0.0 | 3.4 |
| Fe *2p1/2* (2) | 725.8 | 124 | 0.0 | 3.4 |

| Peak | Binding Energy /eV | Height /cps | L-G Mixing | FWHM / eV |
| --- | --- | --- | --- | --- |
| Ni *2p3/2* (1) | 852.7 | 129 | 1.0 | 0.9 |
| Ni *2p3/2* (2) | 855.7 | 205 | 0.2 | 2.2 |
| Ni *2p3/2* (3) | 859.7 | 34 | 0.2 | 2.2 |
| Ni *2p3/2* (4) | 862.2 | 56 | 0.2 | 2.2 |

HAXPES:

| Peak | Binding Energy /eV | Height /cps | L-G Mixing | FWHM / eV |
| --- | --- | --- | --- | --- |
| Fe *2p3/2* (1) | 709.7 | 71 | 0.0 | 3.8 |
| Fe *2p1/2* (1) | 722.9 | 30 | 0.0 | 3.8 |
| Fe *2p3/2* (2) | 712.4 | 27 | 0.3 | 3.6 |
| Fe *2p1/2* (2) | 725.6 | 10 | 0.3 | 3.6 |

| Peak | Binding Energy /eV | Height /cps | L-G Mixing | FWHM / eV |
| --- | --- | --- | --- | --- |
| Ni *2p3/2* (1) | 852.7 | 57 | 0.7 | 1.4 |
| Ni *2p3/2* (2) | 855.0 | 20 | 0.2 | 2.2 |
| Ni *2p3/2* (3) | 857.0 | 9 | 0.2 | 2.2 |
| Ni *2p3/2* (4) | 859.1 | 8 | 0.2 | 2.2 |
| Ni *2p3/2* (5) | 861.7 | 6 | 0.2 | 2.2 |

| Peak | Binding Energy /eV | Height /cps | L-G Mixing | FWHM / eV |
| --- | --- | --- | --- | --- |
| Ni *2p1/2* (1) | 870.1 | 18 | 0.6 | 1.9 |
| Ni *2p1/2* (2) | 872.9 | 9 | 0.2 | 1.9 |
| Ni *2p1/2* (3) | 875.4 | 6 | 0.2 | 1.9 |
| Ni *2p1/2* (4) | 879.0 | 2 | 0.2 | 1.9 |

**Table S5:** XPS/HAXPES fitting parameter of the Fe:Ni = 2:3 NPs.

XPS:

| Peak | Binding Energy /eV | Height /cps | L-G Mixing | FWHM / eV |
| --- | --- | --- | --- | --- |
| Fe *2p3/2* (1) | 710.6 | 920 | 0.0 | 4.1 |
| Fe *2p1/2* (1) | 723.8 | 298 | 0.0 | 4.1 |
| Fe *2p3/2* (2) | 713.0 | 215 | 0.0 | 3.8 |
| Fe *2p1/2* (2) | 726.2 | 82 | 0.0 | 3.8 |

| Peak | Binding Energy /eV | Height /cps | L-G Mixing | FWHM / eV |
| --- | --- | --- | --- | --- |
| Ni *2p3/2* (1) | 852.8 | 682 | 0.1 | 1.4 |
| Ni *2p3/2* (2) | 854.7 | 239 | 0.2 | 2.2 |
| Ni *2p3/2* (3) | 856.5 | 319 | 0.2 | 2.2 |
| Ni *2p3/2* (4) | 858.7 | 152 | 0.2 | 2.2 |
| Ni *2p3/2* (5) | 861.1 | 116 | 0.2 | 2.2 |
| Ni *2p3/2* (6) | 863.3 | 92 | 1.0 | 2.2 |

| Peak | Binding Energy /eV | Height /cps | L-G Mixing | FWHM / eV |
| --- | --- | --- | --- | --- |
| Ni *2p1/2* (1) | 870.0 | 196 | 0.4 | 1.5 |
| Ni *2p1/2* (2) | 871.8 | 22 | 0.2 | 1.9 |
| Ni *2p1/2* (3) | 873.5 | 89 | 0.2 | 1.9 |
| Ni *2p1/2* (4) | 875.0 | 45 | 0.2 | 1.9 |

HAXPES:

| Peak | Binding Energy /eV | Height /cps | L-G Mixing | FWHM / eV |
| --- | --- | --- | --- | --- |
| Fe *2p3/2* (1) | 709.6 | 47 | 0.0 | 4.0 |
| Fe *2p1/2* (1) | 722.8 | 19 | 0.0 | 4.0 |
| Fe *2p3/2* (2) | 712.5 | 215 | 0.0 | 3.6 |
| Fe *2p1/2* (2) | 725.7 | 82 | 0.0 | 3.6 |

| Peak | Binding Energy /eV | Height /cps | L-G Mixing | FWHM / eV |
| --- | --- | --- | --- | --- |
| Ni *2p3/2* (1) | 852.6 | 682 | 0.2 | 1.4 |
| Ni *2p3/2* (2) | 854.1 | 239 | 0.2 | 2.2 |
| Ni *2p3/2* (3) | 856.3 | 319 | 0.2 | 2.2 |
| Ni *2p3/2* (4) | 858.5 | 152 | 0.2 | 2.2 |
| Ni *2p3/2* (5) | 860.6 | 116 | 0.2 | 2.2 |

| Peak | Binding Energy /eV | Height /cps | L-G Mixing | FWHM / eV |
| --- | --- | --- | --- | --- |
| Ni *2p1/2* (1) | 870.0 | 77 | 0.6 | 1.8 |
| Ni *2p1/2* (2) | 873.2 | 16 | 0.0 | 3.0 |
| Ni *2p1/2* (3) | 875.9 | 11 | 0.7 | 3.0 |
| Ni *2p1/2* (4) | 880.4 | 5 | 1.0 | 3.0 |

**Table S6:** XPS/HAXPES Fitting parameter of the pure Ni NPs.

XPS:

| Peak | Binding Energy /eV | Height /cps | L-G Mixing | FWHM / eV |
| --- | --- | --- | --- | --- |
| Ni *2p3/2* (1) | 852.9 | 3183 | 0.4 | 1.2 |
| Ni *2p3/2* (2) | 854.4 | 610 | 0.2 | 2.2 |
| Ni *2p3/2* (3) | 856.5 | 487 | 0.2 | 2.2 |
| Ni *2p3/2* (4) | 859.1 | 227 | 0.2 | 2.2 |
| Ni *2p3/2* (5) | 861.3 | 192 | 0.2 | 2.2 |

| Peak | Binding Energy /eV | Height /cps | L-G Mixing | FWHM / eV |
| --- | --- | --- | --- | --- |
| Ni *2p1/2* (1) | 870.3 | 1056 | 0.2 | 2.1 |
| Ni *2p1/2* (2) | 873.0 | 275 | 0.0 | 2.2 |
| Ni *2p1/2* (3) | 875.3 | 213 | 0.0 | 2.2 |
| Ni *2p1/2* (4) | 878.0 | 138 | 0.2 | 2.2 |
| Ni *2p3/2* (5) | 880.6 | 71 | 0.2 | 2.2 |

HAXPES:

| Peak | Binding Energy /eV | Height /cps | L-G Mixing | FWHM / eV |
| --- | --- | --- | --- | --- |
| Ni *2p3/2* (1) | 853.1 | 593 | 0.2 | 1.2 |
| Ni *2p3/2* (2) | 854.8 | 114 | 0.0 | 2.2 |
| Ni *2p3/2* (3) | 857.2 | 50 | 0.0 | 2.2 |
| Ni *2p3/2* (4) | 859.6 | 42 | 0.2 | 2.2 |
| Ni *2p3/2* (5) | 861.5 | 21.7 | 0.2 | 2.2 |

| Peak | Binding Energy /eV | Height /cps | L-G Mixing | FWHM / eV |
| --- | --- | --- | --- | --- |
| Ni *2p1/2* (1) | 870.4 | 190 | 0.6 | 1.8 |
| Ni *2p1/2* (2) | 874.0 | 16 | 0.0 | 6.1 |
| Ni *2p1/2* (3) | 877.9 | 11 | 0.7 | 3.8 |
